# Transcriptional regulation of porcine PABPN1 gene in adipogenesis

**DOI:** 10.5713/ab.25.0035

**Published:** 2025-06-24

**Authors:** Rong Ru Zhu, Xue Lian Zhao, Ming Hang Chang, Si Qi Yang, Xiao Han Zhang, Ying Ke Liu, Zhi Gang Gu, Xiu Qin Yang

**Affiliations:** 1 College of Animal Science and Technology, Northeast Agricultural University, Harbin, China

**Keywords:** Adipogenesis, PABPN1, Pig, Transcriptional Regulation

## Abstract

**Objective:**

This study aims to reveal the transcriptional regulatory mechanism and effects of poly(A)-binding protein nuclear 1 (PABPN1) on adipogenesis, along with associated polymorphisms.

**Methods:**

Transcription factors were identified using dual-luciferase reporter assay, overexpression techniques site-directed mutagenesis, real-time quantitative polymerase chain reaction (qPCR), electrophoretic mobility shift assays and chromatin immunoprecipitation-qPCR. Preadipocyte differentiation was measured with gain- and loss-of-function, Oil Red O staining and extraction assays. Single nucleotide polymorphisms (SNPs) were identified with direct sequencing of PCR products in the promoter, and the effects of these SNPs on PABPN1 expression were identified with dual-luciferase reporter assay.

**Results:**

CCAAT/enhancer-binding protein (C/EBP) α and β regulate PABPN1 expression by directly binding to its promoter. PABPN1 promotes the preadipocyte differentiation in pigs. Three SNPs were identified, with the Haplotype GCC mutation significantly increasing the promoter activity of PABPN1.

**Conclusion:**

PABPN1 promotes the preadipocyte differentiation as a downstream gene of C/EBP α and β. Haplotype GCC may serve as a molecular marker for selection of fat traits in pigs.

## INTRODUCTION

Poly(A)-binding protein (PABP) is a kind of protein binding to the poly(A) tails of mRNA [1]. They were classified into two groups according to the localization in cells: proteins located in the nucleus were named PABP nuclear 1 (PABPN1) and PABPN1-like, while those in cytoplasm were named PABPC1 (PABP cytoplasmic 1) and its homologous proteins [2,3]. PABPN1 is ubiquitously expressed and highly conserved among mammals [4]. There are few differences in the amino acid sequences among PABPN1s from human, mouse and pig.

As a nuclear poly(A) binding protein, PABPN1 has been implicated in various cellular steps of mRNA metabolism [5]. PABPN1 is crucial for mRNA polyadenylation. Studies have shown that PABPN1 first bound nascent poly(A) tails directly and stimulate poly(A) polymerase (PAP) activity and processivity through stabilizing the interaction of PAP with RNA, then it could stop the elongation of poly(A) tail, controlling the length of poly(A) tail to 200–300 nucleotides [6–9]. Through binding to the poly(A) tails, PABPN1 could influence intron excision and mRNA decay [10,11]. PABPN1 also contributes to exporting mature mRNA to the cytoplasm [5]. However, for an intron retained mRNA, PABPN1 can prevent its nuclear export and translation, thus playing a role in gene expression via intron retention [12].

Recently, alternative polyadenylation has attracted widespread attention due to its importance in post-transcriptional regulation of gene expression. It has been revealed that more than about 79% of protein-coding genes and 66% of long noncoding RNA (lncRNA) genes have>1 poly(A) site in mice [13]. Generally, proximal poly(A) sites are weaker because non-canonical poly(A) signals (PASs), variants to the canonical PAS, were enriched in the sites. The binding of PABPN1 on the non-canonical PASs could compete with the recruitment of cleavage and polyadenylation specificity factor on these weak PASs, thus repressing the usage of proximal poly(A) site [9,14]. Decreases in PABPN1 levels enhanced the usage of non-canonical PASs, resulting in extensive shortening of 3’ untranslated region, which prevents the post-transcriptional regulation by microRNAs targeting sequences downstream of the proximal PASs.

Studies also highlight the role of PABPN1 in various physiological processes including oocyte development [15], maternal mRNA clearance [16,17], cell proliferation [18], apoptotic [19] and differentiation [20]. However, knowledge on the regulatory mechanism of PABPN1 expression is limited, and the role of PABPN1 in fat accumulation remains to be revealed. Pig is the most important agricultural animals in China. The total production and consumption of pork accounts for more than 50% of China’s total meat production. With the improvement of people’s living standard, more attention was paid on meat quality. Fat deposition is one of factors determining meat quality and intramuscular fat content has been associated with meat quality directly. To reveal the relationship between PABPN1 and fat deposition, this study was designed to clarify the transcription factors (TFs) associated with fat deposition and to further reveal its role in the process. Additionally, single nucleotide polymorphisms (SNPs) influencing the expression of PABPN1 gene were analyzed. The results will contribute to further clarifying the transcriptional regulation mechanism of PABPN1 gene and to manipulating the expression of PABPN1 in cells. Also, the results provide a potential marker for selecting fat accumulation in pig breeding.

## MATERIALS AND METHODS

### Animals, nucleic acids, and cDNA

Min pigs, a Chinese indigenous pig breed, were obtained from the Institute of Animal Husbandry, Heilongjiang Academy of Agricultural Sciences, Harbin, China. For preadipocyte isolation and cDNA synthesis, three Min pigs at age of 30-day were used, and back fat tissues were sampled immediately after the pigs were slaughtered. Fresh tissues were used for preadipocyte isolation, and those for cDNA synthesis were stored at −80°C after snap frozen in liquid nitrogen. For polymorphism analysis, a total of 11 adult Min pigs were used and ear tissues were collected. Total RNA was extracted with TRIzol reagent (Invitrogen). Genomic DNA was obtained with normal phenol-chloroform method. cDNA was synthesized with HiScript III 1st Strand cDNA Synthesis Kit (+gDNA wiper) (Vazyme). All procedures of animal treatment were approved by the Laboratory Animal Welfare and Ethics Committee of Northeast Agricultural University.

### Preadipocyte culture, differentiation, and Oil Red O Staining

Preadipocytes were obtained from backfat tissues of 30-day-old Min pigs as described previously [21]. Briefly, backfat tissues were first digested with 0.1% type I collagenase (Invitrogen), and then cultured in DMEM/F12 (Dulbecco’s Modified Eagle’s Medium/Nutrient Mixture F-12) medium supplemented with 10% FBS (fetal bovine serum: Gibco) and 1% penicillin-streptomycin (Invitrogen) at 37°C with 5% CO_2_. When the cells reached contact inhibition, DMEM/F12 medium containing 10% FBS, 1 μmol/L dexamethasone, 0.5 mmol/L 3-isobytyl-1-methylxanthine and 5 μg/mL insulin was used to induce differentiation. At two days post induction, the cells were transferred to DMEM/F12 medium containing 10% FBS and 5 μg/mL insulin to maintain the differentiation for another six days. The medium was changed every 48 h. The mature adipocyte was stained with Oil Red O (Leagene), and the results were viewed and photographed with a light microscope (Analytik Jena). At the same time cellular Oil Red O was extracted with isopropanol, and the triglyceride content was quantified with optical absorbance (OD) at 510 nm.

### Plasmids and mutagenesis

Fragments spanning −1,576 to −90 bp of porcine PABPN1 promoter were amplified from genomic DNA. Polymerase chain reaction (PCR) was performed with *Ex* Taq (TaKaRa). The resultant products were inserted into pGL3-basic at Kpn I and Hind III sites to construct reporter genes, 15F/R. Here, the first nucleotide of start codon was designated as +1. The 5’ truncated fragments were then amplified using 15F/R as templates, and the reporter genes were constructed. Over-lap extension PCR was performed to mutate the fragments as described previously [21] and. Briefly, the mutation was first introduced into the end of two over-lap fragments, respectively, with primers, then the two fragments were spliced to introduce the mutation into the inner of the fragments. High-fidelity Pfu DNA polymerase (Trans Gene) was used in the over-lap extension PCR. The mutant fragment obtained were inserted into pGL3-basic at Kpn I and Hind III sites to construct mutant type reporter genes.

The complete coding sequences (CDS) of CCAAT-enhancer binding protein (C/EBP) α and β were amplified using cDNA template obtained from fat tissues, respectively, and inserted into pCMV-HA vector at EcoR I and Xho I sites. Overexpression vector of C/EBPα constructed with pcDNA3.1+ was obtained previously [21]. Plasmids pcDNA3.1-C/EBPα and pCMV-HA-C/EBPα were used for dual-luciferase reporter and electrophoretic mobility shift assay (EMSA), respectively. Plasmids overexpressing and siRNA against PABPN1 were obtained previously [18]. All primers here were synthesized by Genesoul Tech and listed in Supplements 1–3.

### Transient transfection

Transient transfection was carried out with lipofectamine 2000 (Invitrogen) according to the manufacturer’s instructions. In PK-15 cells, at 48 h post-transfection the cells were collected for dual-luciferase reporter assay or real-time quantitative PCR (qPCR). In preadipocytes, at 24 h post-transfection the cells were induced to differentiate and the induction time was indicated in Results. The preadipocytes were then used for Oil Red O staining or qPCR.

### Dual-luciferase reporter analysis

PK-15 cells were cultured as described previously [21]. Each reporter gene was transfected into the cells individually or together with overexpression vector. *Renilla* luciferase reporter, pRL-TK, was used as internal reference. At 48 h post transfection, luciferase activities were measured with dual-luciferase reporter gene assay kit (Beyotime), and relative luciferase activity was calculated as a ratio of firefly to *Renilla* luciferase activities.

### Real-time quantitative polymerase chain reaction

qPCR was performed with ChamQ Universal SYBR qPCR Master Mix (Vazyme) according to the protocol of manufacturer. The relative expression level of target gene was calculated with 2−ΔΔCt method with β-actin as a reference [22]. Primers for C/EBPα and C/EBPβ were synthesized by Genesoul Technology and those for PABPN1 and β-actin were obtained previously [18]. Primer sequences were listed in Supplement 4.

### Western blotting

PK-15 cells, cultured in six-well plates, were transfected with plasmid pCMV-HA-C/EBPα and pCMV-HA-C/EBPβ, respectively, for 48 h. Total protein was isolated with RIPA buffer (Beyotime) containing a protease inhibitor (Invitrogen). BCA protein assay kit (Beyotime) was used to quantify the protein. 25–30 μg total protein was loaded on SDS-polyacrylamide gel and separated at 80 V. The protein was then transferred onto a polyvinyl difluoride membrane (Millipore) and incubated with anti-HA tag (1:5,000 dilution; Abmart) and anti-β-actin (1:3,000 dilution; Abmart) primary antibodies. Goat anti-mouse IgG (1:20,000 dilution; LI-COR) was used as secondary antibody. The results were visualized on UVP ChemStudioTM PLUS touch (Analytik Jena).

### Electrophoretic mobility shift assay

EMSA was performed as described previously [21]. Briefly, the biotin-labeled gene-specific binding probes, unlabeled specific and mutant competitors were synthesized by General Biol. Briefly, overexpression vector was transfected into HEK-293T cells using lipofectamine 2000 (Invitrogen). At 48 h post transfection, nuclear extracts were isolated with the kit purchased from Solarbio. Incubation was performed with Chemiluminescent kit (Beyotime) according to the manufacturer’s instructions. The biotin-labeled probe was incubated with 20 μg nuclear extracts singly or together with mutant competitor. For co-incubation of biotin-labeled probe and unlabeled specific competitor, the unlabeled probes were first incubated with nuclear extracts for 10 min, and then the labeled ones were added. Electrophoresis was performed on 6.5% polyacrylamide gel for 1.5 h at 90 V. After transferred to nylon membrane (Beyotime), the gels were viewed on Azure c300 Gel Imaging System (Zzure Biosystems). The probe sequences were given in Supplement 5.

### Chromatin immunoprecipitation-real-time quantitative polymerase chain reaction

PK-15 cells were cultured until 70%–80% confluence, and transfected for 48 h. The Chromatin immunoprecipitation (ChIP)-qPCR was performed with SimpleChIP Enzymatic Chromatin IP Kit (Cell Signaling) according to the manufacturer’s instructions. Briefly, the crosslinking reaction was performed in 1% formaldehyde for 10 min at room temperature, and terminated with 10× glycine solution. The cells were collected using 2 mL ice-cold PBS solution and 200× Protease Inhibitor Cocktail, and nuclear pellet was isolated at 2,000×g for 5 min at 4°C. Ultrasonic Homogenizer was used to shiver DNA to a length of about 150–900 bp, and the products were blotted with anti-HA tag (Abmart) overnight at 4°C with rotation. The immunoprecipitated DNA was quantified with qPCR, and the primers were listed in Supplement 6.

### RNA sequencing

RNA sequencing (RNA-seq) was performed to identify genes regulated by PABPN1 in porcine preadipocytes. SiRNAs against PABPN1 and negative control (NC) sequences were transfected into cells, respectively, with lipofectamine 2000 (Invitrogen), each with triplicate. At 24 h post transfection, the cells were induced to differentiate for 48 h. RNA was isolated and detected with NanoDrop 2000 (IMPLEN), Qubit 2.0 (Thermo Fisher Scientific), and Agilent 2100 to examine the purity, concentration, and integrity of the sample. Qualified RNA was subjected to library construction and a total of six libraries (NC = 3, siPABPN1 = 3) were obtained. RNA-seq was performed on the NovaSeq6000 PE150 platform (Illumina) at Allwegene Technologies.

The clean reads were mapped to the pig reference genome (S. scrofa 11.1_release 109) using HISAT2 (v2.0.4) [23]. The mapped reads were assembled into transcripts with StringTie (v2.2.1) [24]. FPKM (Fragments Per Kilobase of transcript per Million fragments mapped) [25] was used to measure the gene expression level, and genes with FPKM≥0.5 in at least three biological repetitions were used for further analysis. Differentially expressed genes (DEGs) were identified using DESeq2 [26] in cells knocking down PABPN1 compared to that transfected with NC sequences, and the threshold was set as Fold Change (FC)≥1.2 and p<0.05. Gene Ontology (GO) and Kyoto Encyclopedia of Genes and Genomes (KEGG pathway) was performed as described previously [27].

### Single nucleotide polymorphism analysis

Fragments spanning −1,094 to −90 were amplified with genomic DNA template. The products were sequenced directly by Genesoul Technology to analyze SNPs. The sequences obtained were aligned by using SnapGene (v6.0.2) to identify SNPs. At the same time manual inspection of the diagram was performed with the Chromas software to confirm the SNPs and the genotype. Linkage disequilibrium analysis was performed with the online program SHEsis ( http://analysis.bio-x.cn/myanalysis.php, accessed on 12 September 2024).

### Statistical analysis

All experiments were performed in three independent experiments, each with three biological repetitions. Statistical analyses were carried out with GraphPad Prism (v9.5.1). Difference between two groups was analyzed with unpaired t-test, while that among multiple groups was analyzed with one-way ANNOVA. Multiple comparison was performed using default method in ANNOVA program of GraphPad Prism (v9.5.1).

## RESULTS

### C/EBPα upregulates the transcription of porcine PABPN1 by directly binding to the motif

**Figure 1:**
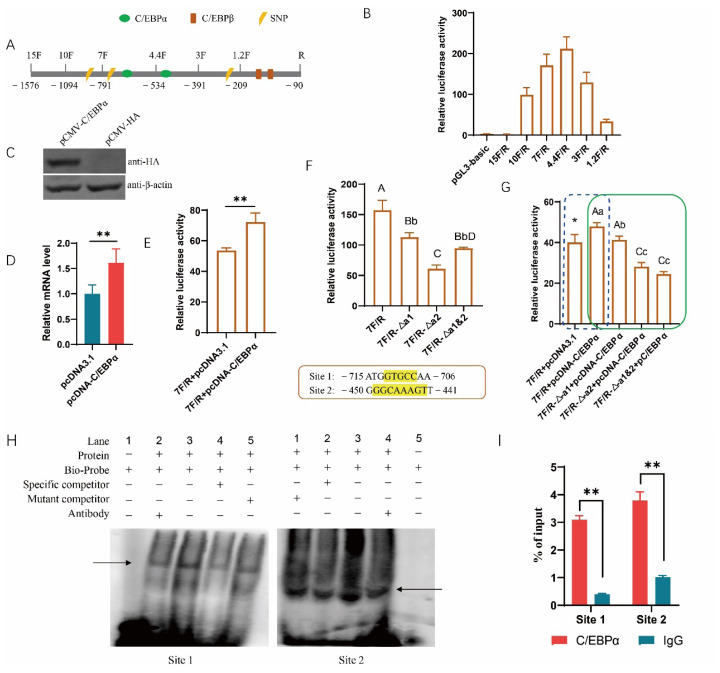
C/EBPα upregulates the expression of PABPN1 through directly binding to the promoter. (A) Schematic structure of fragments inserted into pGL3-basic. F, forward primer; R, reverse primer; vertical line indicates the position of the 5’ end of the forward primer or the 3’ end of the reverse primer; (B) Characterization of promoter activity. 15F/R, 10F/R, etc. indicate reporter genes containing the fragment amplified with the primer pair; (C) Efficiencies of plasmids overexpressing C/EBPα as revealed with western blotting; (D) Effects of C/EBPα on the expression of endogenous PABPN1 as revealed with real-time quantitative PCR; (E) Effects of ectopic C/EBPα on activity of PABPN1 promoter as revealed with dual-luciferase reporter assay; (F) Effects of putative motifs for C/EBPα on the activity of PABPN1 promoter. The position and nucleotides of the two putative binding sites for C/EBPα, Site 1 and Site 2, were given below, and the nucleotides deleted in the mutants were marked with yellow. Δα1 or Δα2 indicate the Site 1 or Site 2 was deleted in the reporter gene, while Δα1&2 indicates the two motifs were deleted simultaneously; (G) Deletion of binding motif abolishes the promoting effects of ectopic C/EBPα on the activity of PABPN1 promoter; (H) Electrophoretic mobility shift assay (EMSA) confirms the binding of C/EBPα to the motifs. The arrow indicates the DNA-protein complex; (I) ChIP-qPCR assay confirms the binding of C/EBPα to the motifs. * p<0.05, ** p<0.01; ^A–D, a–c^ The bar with different letter indicates the difference is significant, and lowercase and uppercase indicate p<0.05 and p<0.01, respectively. SNP, single nucleotide polymorphism; PCR, polymerase chain reaction.

To investigate the transcriptional regulation of porcine PABPN1 in detail, the 5’ flanking region were first subjected to bioinformatic analysis using online software. It is interesting that C/EBPα and β, two well-known TFs for fat accumulation, were predicted in the promoter of PABPN1. Each of the TFs has two putative binding sites (Figure 1A). To investigate the role of C/EBPα and β in PABPN1 transcription, reporter genes containing serial 5’ flanking region of PABPN1 were constructed to identify the promoter activity (Figure 1A). It was revealed that fragments spanning −1,094 to −90 bp have promoter activity with the highest level in region −534 to −90 bp, and the core promoter was situated in −209 to −90 bp; while the longest fragment (−1,576 to 90 bp) did not show activity to drive the expression of firefly luciferase gene, indicating there is motif inhibiting the transcription (Figures 1A, 1B).

Two plasmids overexpressing C/EBPα, pcDNA3.1-C/EBPα and pCMV-C/EBPα, were used here. The efficiencies of pcDNA3.1-C/EBPα were characterized previously [21]. Western blotting showed that the plasmids pCMV-C/EBPα were constructed successfully (Figure 1C). We first focused on the role of C/EBPα in PABPN1 transcription. The mRNA level of PABPN1 was significantly increased by ectopic C/EBPα in PK-15 cells (p<0.01; Figure 1D). C/EBPα overexpression significantly enhanced the activity of PABPN1 promoter as revealed by reporter gene analysis (p<0.01; Figure 1E). Absence of any of the two putative binding sites, mapped to −715 to −706 and −450 to −441 (named Site 1 and Site 2), respectively, resulted in significant decreases in the promoter activities compared to the wild type fragment. Compared to site 1, Site 2 has a more powerful impact on the promoter activity (Figure 1F). Additionally, deleting the motifs, either singly or simultaneously, abolished the promoting effects of ectopic C/EBPα on promoter activity (Figure 1G).

At the same time, EMSA was used to analyze the binding ability of C/EBPα to the motifs. It was revealed that the DNA-protein complexes could be formed clearly in groups containing biotin-labeled probe in both sites, addition of mutant competitor did not affect the formation of the complexes, while specific competitor weaken the complexes. The addition of antibody against HA tag also led to weakness of the complexes (Figure 1H). Furthermore, ChIP-qPCR showed that C/EBPα enriched significantly in the both motifs of PABPN1 promoter (Figure 1I). These results indicated C/EBPα regulated the expression of PABPN1 by directly binding to the promoter.

### C/EBPβ is also a transcription factor of porcine PABPN1

**Figure 2:**
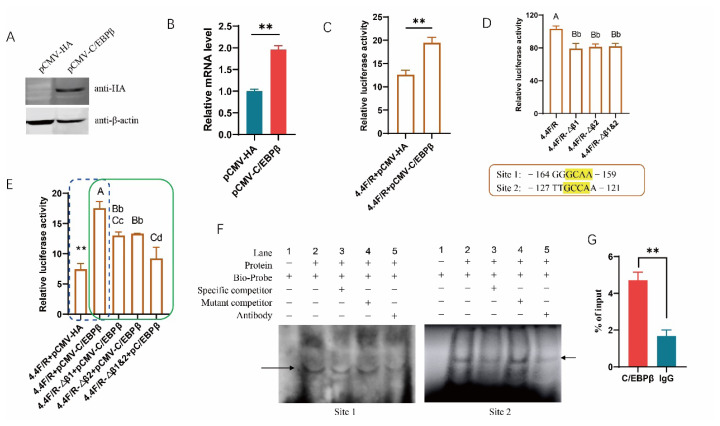
C/EBPβ upregulates the expression of PABPN1 through directly binding to the promoter. (A) Efficiencies of plasmids overexpressing C/EBPβ as revealed with western blotting; (B) Effects of C/EBPβ on the expression of endogenous PABPN1 as revealed with real-time quantitative PCR; (C) Effects of ectopic C/EBPβ on activity of PABPN1 promoter as revealed with dual-luciferase reporter assay; (D) Effects of putative motifs for C/EBPβ on the activity of PABPN1 promoter. The position and nucleotides of the two putative binding sites for C/EBPβ, Site 1 and Site 2, were given below, and the nucleotides deleted in the mutants were marked with yellow. Δβ1 or Δβ2 indicate the Site 1 or Site 2 was deleted in the reporter gene, while Δβ1&2 indicates the two motifs were deleted simultaneously; (E) Deletion of binding motif abolishes the promoting effects of ectopic C/EBPβ on the activity of PABPN1 promoter; (F) EMSA confirms the binding of C/EBPβ to the motifs. The arrow indicates the DNA-protein complex; (G) ChIP-qPCR assay confirms the binding of C/EBPβ to the motifs. ** p<0.01; ^A–C, a–d^ The bar with different letter indicates the difference is significant, and lowercase and uppercase indicate p<0.05 and p<0.01, respectively. PCR, polymerase chain reaction.

Two putative binding sites for C/EBPβ were mapped to −164 to −159 and −127 to −121 (named Site 1 and Site 2) in the promoter of porcine PABPN1, respectively. Western blotting showed that the plasmids C/EBPβ were constructed successfully (Figure 2A). We first showed that ectopic C/EBPβ increased the mRNA level of PABPN1 in PK-15 cells (p<0.01; Figure 2B). Dual-luciferase reporter gene analysis revealed that ectopic C/EBPβ led to significant increases in the promoter activity of PABPN1 (p<0.01; Figure 2C). Deletion of any one of the sites resulted in significant decreases in the promoter activity, but no synergistic effects were found in mutants absent of two sites (Figure 2D). As expected, deletion of the sites significantly decreased the promoting effects of ectopic C/EBPβ on PABPN1 promoter activity, and no differences were found between mutants deleting single sites (Figure 2E). EMSA assay showed that C/EBPβ could bind to Site 2 directly as complexes were formed clearly between nuclear extracts and biotin-labeled probes in both sites, and mutant competitor had no effects on the formation of complexes, while specific competitor weakened the complexes robustly. Additionally, antibody against HA tag significantly decreased the complex intensity (Figure 2F). Because the two sites are close to each other, ChIP-qPCR was performed on the two sites simultaneously, and the results showed that C/EBPβ were significantly enriched in the region (Figure 2G). However, the binding of C/EBPβ on Site 1 were not shown clearly by EMSA assay, and we did not perform ChIP-qPCR on the site individually. We cannot confirm whether it is a genuine site for C/EBPβ. Nevertheless, we made clear that there was at least one binding site for C/EBPβ in the promoter of PABPN1, and that C/EBPβ could regulate the transcription of PABPN1 by directly binding to the promoter.

### PABPN1 is involved in adipogenesis

**Figure 3:**
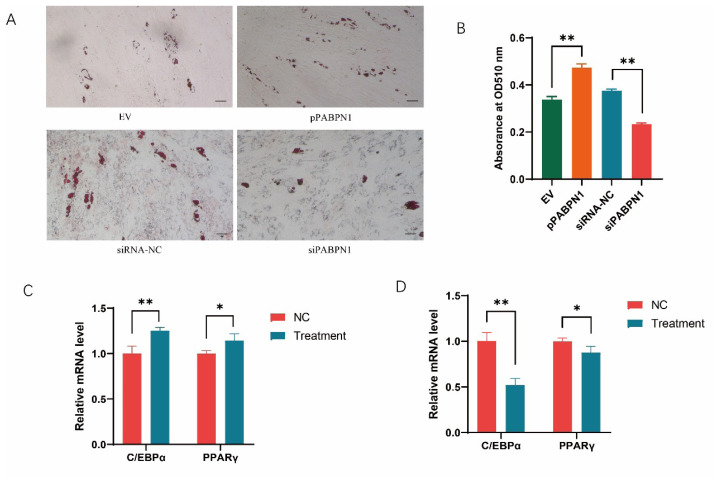
PABPN1 promotes preadipocyte differentiation. (A) PABPN1 promotes adipogenesis as revealed by Oil Red O staining assay. The bar is 100 μm; (B) PABPN1 improves triglyceride contents as revealed with Oil Red O staining extraction assay; (C, D) Effects of PABPN1 overexpression (C) and knockdown (D) on mRNA expression of adipogenic marker, C/EBPα and PPARγ. * p<0.05, ** p<0.01.

It is well known that C/EBPα and β are key regulators of adipogenesis, suggesting a role of PABPN1 in the process. We then analyzed the effects of PABPN1 on adipogenesis through gain- and loss-of-function assays in primary preadipocytes isolated from subcutaneous fat tissues of 30-d-old pigs. Plasmids overexpressing and siRNA against PABPN1 were obtained successfully previously [18]. The ectopic PABPN1 promoted the differentiation of preadipocytes, while knockdown of PABPN1 inhibited it, as revealed by Oil Red O staining at 6-days post-induction (Figure 3A). Consistently, triglyceride contents were increased by overexpressing PABPN1, and decreased by interfering PABPN1, in porcine preadipocytes (Figure 3B). At the same time the expression of adipogenesis marker genes, C/EBPα and peroxisome proliferator-activated receptor (PPAR) γ, was significantly increased by ectopic PABPN1, and decreased by knockdown of PABPN1 (Figures 3C, 3D).

**Figure 4:**
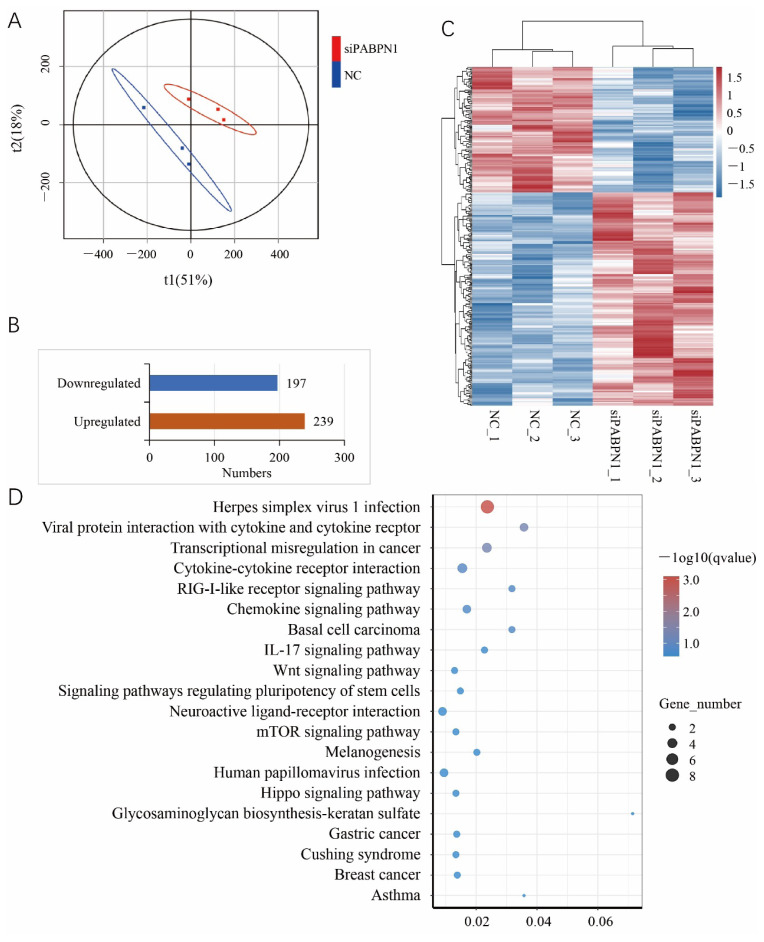
Characterization of genes regulated by PABPN1 with RNA-sequencing in preadipocytes knocking down PABPN1. (A) Principle component analysis; (B) Statistics of differentially expressed genes (DEGs); (C) Heatmap of DEGs; (D) Top 20 KEGG pathways enriched by DEGs.

To further reveal the role of PABPN1 in adipogenesis, RNA-seq was performed on preadipocytes knocking down PABPN1 (Supplement 7). Principle component analysis showed that the RNAi and NC groups were separated well, and there were small differences between samples in the same group (Figure 4A). A total of 436 DEGs including 239 upregulated and 197 downregulated were identified in preadipocytes knocking down PABPN1 compared to NC groups (Figures 4B, 4C; Supplement 8). There are a lot of TFs among the DEGs including three members of nuclear receptor subfamily 4 group A, two members of homeobox family, three members of zinc finger protein, signal transducer and activator of transcription 3, etc. Additionally, known regulators for fat deposition such as lipoprotein lipase, apelin, secreted frizzled related protein 2, notch receptor 3, etc. were also differentially expressed. However, C/EBPα and PPAR γ were not found in DEGs, which might be caused by the fact that the induction time was limited. A series of KEGG pathways were significantly enriched by DEGs (q<0.05). Among the top 20 pathways enriched five have been involved in fat accumulation including RIG-I-like receptor signaling pathway, Wnt signaling pathway, IL-17 signaling pathway, mTOR signaling pathway and Hippo signaling pathway according to literature retrieval. Among them RIG-I-like receptor signaling pathway and IL-17 signaling pathway were the top 2 significantly enriched. Additionally, pathways involved in human diseases were also significantly enriched by DEGs (Figure 4D).

### Single nucleotide polymorphisms affect the expression of PABPN1 in the promoter

**Figure 5:**
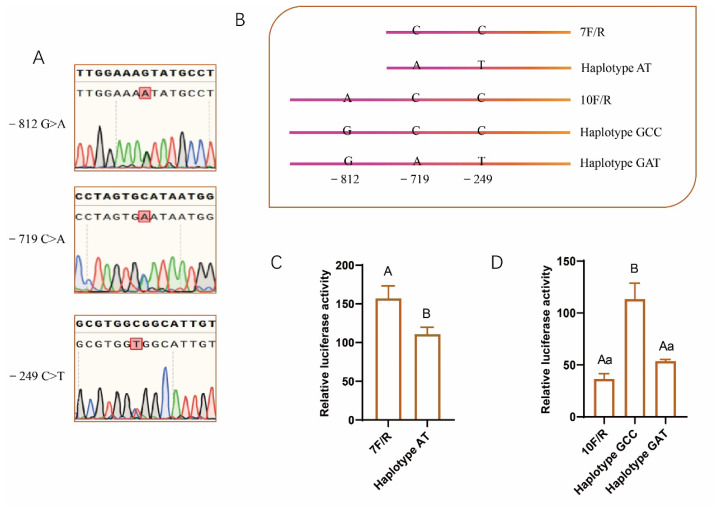
Haplotype variation is associated with the expression of PABPN1. (A) Sequencing diagram of the three single nucleotide polymorphisms (SNPs) exemplified with heterozygotes; (B) Schematic structure of mutant fragments used to construct reporter genes; (C) Effects of two tightly linked SNPs on the promoter activity; (D) Characterization of haplotype mutant associated with promoter activity. ^A,B,a^ The bar with different letter indicates the difference is significant, and lowercase and uppercase indicate p<0.05 and p<0.01, respectively.

Three SNPs, −812A>G, −719C>A and −249C>T, were identified in the promoter of PABPN1 by directly sequencing of PCR products (Figure 5A). Genotyping analysis showed that these SNPs occurred frequently in Min pigs. Among 11 Min pigs analyzed, the allele frequencies of G, A and T in sites −812, −719 and −249 were 13.64%, 77.3% and 77.3%, respectively (Table 1). SNPs −719C>A and −249C>T are in complete linkage disequilibrium with the parameter D’ = 1.0 and r^2^ = 1.0 as revealed by the online software SHEsis.

The effects of the SNPs on the expression of PABPN1 were analyzed with dual-luciferase reporter gene. We first double mutated CC to AT in the reporter gene 7F/R to analyze the effects of the two linkage SNPs (Figure 5B). Dual-luciferase reporter assay showed that the mutation significantly decreased the promoter activity (p<0.01; Figure 5C), indicating Haplotype AT had an inhibitory role on the expression of PABPN1 gene.

Next, the role of SNP −812A>G was analyzed. Mutagenesis was conducted on the reporter gene 10F/R containing A, C and C at sites −812, −719 and −249, respectively, and named Haplotype ACC. Haplotype GCC was obtained by changing nucleotide A in site −812 to G (Figure 5B). We found that this single mutation significantly increased luciferase activity (p<0.01; Figure 5D). Then haplotype GAT were created (Figure 5B), and no differences were found in luciferase activities between reporter genes containing Haplotype ACC and Haplotype GAT (p>0.05; Figure 5D). Compared to haplotype GCC, the activity of haplotype GAT was significantly decreased (p<0.01; Figures 5B, 5D). Thereafter we characterized a haplotype promoting the expression of PABPN1 gene, Haplotype GCC.

## DISCUSSION

PABPN1 is important for mRNA stability, expression and nuclear exportation, and thereafter crucial for various physiological and pathological processes. It is of significance to reveal the underlying regulatory mechanisms of PABPN1 and to further explore its role in cells. Here, we found that PABPN1 was regulated by two well-known adipogenic TFs, C/EBPα and β, and promoted adipogenesis in pigs. Additionally, a haplotype mutation increasing the expression of PABPN1 gene was identified. The results will contribute to further reveal the role and regulatory mechanisms of PABPN1 in adipogenesis.

Studies on PABPN1 mainly focused on its role in physiological processes and the pathogenesis of diseases. Dysregulation of PABPN1 was associated with various diseases such as tumorigenesis and progression [28–30], muscle wasting [31], myogenic disability [32], muscle degeneration [33], etc. It is important for controlling the PABPN1 expression to reveal the regulatory mechanisms underlying the expression. To the best of our knowledge, only cAMP-response element binding protein (CREB) was thus far identified as the TF of PABPN1 [18,27]. Efforts need to be made to reveal the factors regulating PABPN1 expression. Here, we first analyzed the transcriptional regulatory mechanism of PABPN1 in detail by using online software. Among the TFs predicted C/EBPα and β, two well-known important adipogenic regulator [34], attracted our interest. Considering the fact that the established TF of PABPN1, CREB, has been involved in adipogenesis in pigs [35], we hypothesized that PABPN1 might play a role in adipogenesis. Thereafter, we characterized the effects of C/EBPα and β on the transcriptional expression of PABPN1 with molecular biology techniques. As expected, both TFs regulated the expression of PBBPN1 by directly binding to the promoter. It is revealed that each of the TFs, C/EBPα or β, has two active binding motifs as revealed by site-directed deletion, overexpression, EMSA and ChIP-qPCR experiments.

C/EBPα, together with PPARγ, is characterized as the master TFs for terminal adipocyte differentiation [36]. C/EBPα is essential for fat accumulation by regulating the expression of various genes including fatty acid binding protein 4 [18,37] and acetoacetyl-CoA synthetase [38]. The absence of C/EBPα suppresses the development of white adipose tissue [39]. C/EBPβ is an important early regulator of adipogenesis. It is induced rapidly in the presence of adipogenic stimuli and responsible for transactivating the expression of C/EBPα and PPARγ by directly binding to their promoters [40]. Disruption of the C/EBPβ gene resulted in decreased fat mass in mice [41]. These results indicate both C/EBPα and β are essential for terminal adipocyte differentiation and fat accumulation. The involvement of the two TFs in the transactivation of PABPN1 further highlights the role of PABPN1 during adipogenesis. Through gain- and loss-of-function assays it was first revealed that PABPN1 promoted preadipocyte differentiation. Although many factors have been involved in preadipocyte differentiation including a cascade of multiple TFs, major genes and epigenetic modifiers [42–44], molecular details of adipogenesis remain to be identified. The results obtained here replenish the genetic database responsible for adipogenesis. However, we did not reveal the mechanisms through which PABPN1 regulates preadipocyte differentiation here. Further efforts are needed to clarify the question.

Both the increases in adipocyte number and size, that is, adipocyte hyperplasia and hypertrophy, are major contributors to fat development. We have revealed that PABPN1 inhibited the proliferation of preadipocytes by suppressing cell cycle progression [18]. Cell cycle arrest and induced expression of C/EBPα and PPARγ are equally important to terminal adipogenic differentiation. Both of the events jointly result in formation of fat droplets in cytosol [45,46]. PPARγ activation tightly accompanies cell cycle exit [45,47]. Combined with the two results obtained here and previously [18], we made sure that PABPN1 regulated fat accumulation by repressing preadipocyte proliferation and enhancing preadipocyte differentiation in pigs.

We next identified important genes regulated by PABPN1 during preadipocyte differentiation with RNA-seq. KEGG enrichment was used to evaluate the role of DEGs and it was found RIG-I-like receptor signaling pathway and IL-17 signaling pathway were the top 2 pathways related to adipogenesis. It is well-known that both RIG-I-like receptor and IL-17 are regulators of innate immune responses and extensively involved in pathogenesis of diseases. Compared to RIG-I-like receptor signaling pathway, IL-17 signaling pathway are more frequently involved in adipogenesis [48–50]. Studies even revealed that IL-17A could promote transdifferentiation of C2C12 cells, the mouse myoblast cells, into adipocytes by enhancing the expression of PPARγ through C/EBPβ signaling [51]. While there is only one study clearly indicated that family with sequence similarity 13 member A inhibited intramuscular preadipocyte differentiation through RIG-I Receptor Signaling Pathway [52]. Our results further highlight the role of RIG-I Receptor Signaling Pathway in regulating adipogenesis.

PABPN1 is highly conserved among species. At the amino acid level, there are few differences among polypeptides from human, mouse and pig. Mutations of PABPN1 caused by expansions of (GCN) triplet at the 5’-end of CDS result in with Oculopharyngeal muscular dystrophy [53,54]. SNPs have not been identified in PABPN1 gene as yet. Here, a total of three SNPs were first identified in the promoter of PABPN1 gene in Min pigs, a Chinese local pig breed with characteristics of high fat content, by using direct sequencing of PCR products. It was further showed that the SNPs played a role in regulating the gene expression as revealed by dual-luciferase assay. Additionally, the haplotype mutation, Haplotype GCC, led to significant increases in the promoter activity. Combined with the role of PABPN1 in adipogenesis, the Haplotype GCC should contribute to promoting preadipocyte differentiation by enhancing the expression of PABPN1. Further analysis will reveal the potential of Haplotype GCC as molecular marker for selecting fat deposition.

## CONCLUSION

The effects of PABPN1 on preadipocytes differentiation were initially revealed. It was found that C/EBPα and β, two important regulators of adipogenesis, regulated the expression of PABPN1 by directly binding to the promoter. Further analyses confirmed that PABPN1 promoted preadipocyte differentiation, and important KEGG pathways through which PABPN1 regulated adipogenesis were identified. Additionally, a haplotype mutation in the promoter, Haplotype GCC, was found to increase the expression of PABPN1. The results will contribute to further revealing the mechanisms underlying the regulation of PABPN1 in fat accumulation, and to characterize molecular marker for selecting fat trait.

## Figures and Tables

**Table 1:** Single nucleotide polymorphisms of PABPN1 promoter among 11 Min pigs

Locus	Allele frequency (%)		Genotype frequency (%)	
−812 A>G	A	86.36	AA	72.7
			AG	27.3
	G	13.64	GG	
−719 C>A	C	22.7	CC	0
			CA	45.5
	A	77.3	AA	54.5
−249 C>T	C	22.7	CC	0
			CT	45.5
	T	77.3	TT	54.5

## SUPPLEMENTARY MATERIAL

**Supplement 1.** Primers used for reporter gene construction. **Supplement 2.** Primers used for amplification of coding sequences. **Supplement 3.** Primers used for site-directed mutagenesis. **Supplement 4.** Primers used for real-time quantitative PCR. **Supplement 5.** Oligonucleotides used for EMSA. **Supplement 6.** Primers used for ChIP-qPCR.

**Supplement 7.** Overview of RNA-seq data.

**Supplement 8.** Differentially expressed genes.
