# Supplementary material for: Transcriptional regulation of porcine PABPN1 gene in adipogenesis
Source: Anim Biosci. 2025 Jun 24;38(12):2584–96. doi: 10.5713/ab.25.0035 (PMC12580747; doi:10.5713/ab.25.0035)
Supplement: Supplementary file 1 [file ab-25-0035-Supplementary-1-6.pdf]

Supplements 1-6 Oligo nucleotides used in this study

Supplement 1. Primers used for reporter gene construction.

Names	Sequences (5'-3')	Purpose
15F	GGTACCGGCTCTGAAACCGGTAGC	Construction of wild
10F	GGTACCAAAAGGACTGACTGCATGTAGAGG	type gene
7F	GGTACCGCACGGTTAGAACTTGTGACTGG	
4.4F	GGTACCCCAACAGTATCAAGATGGCGGC	
3F	GGTACCGCGTTTGAGACTGGGCCACT	
1.2F	GGTACCCGTTGATTGACAGACCGATT	
R	AAGCTTCAATGGACTCCTAGCTCGCC	
CEBPα-1-mut-F	GCATAATGGTGAACCTGAGTGG	Deleting CEBPα motif
CEBPα-1- mut-R	CCACTCAGGTTCACCATTATGC	
CEBPα-2-mut-F	AGTTCGTTTGGGTCACCTGTCAC	
CEBPα-2- mut-R	GTGACAGGTGACCCAAACGAACT	
CEBPβ-1-mut-F	GAATTTGGATCCCCCGCCTTA	Deleting CEBPβ motif
CEBPβ-1-mut-R	TAAGGCGGGGGATCCAAATTC	
CEBPβ-2-mut-F	GGTCTTATTTGATTAGTAATATTC	
CEBPβ-2-mut-R	GAATATTACTAATCAAATAAGACC	

Enzyme sites were underlined.

Supplement 2. Primers used for amplification of coding sequences.

Names	Sequences (5'-3')
CEBPα-F	GGGGTACCTTATGGAGTCGGCCGACTTCTAC
CEBPα-R	CGGAATTCTCAAGCGCAGTTGCCCATGG
CEBPβ-F	CCGGAATTCTTGCCACCATGCAACGCCTGGTGGCCT
CEBPβ-R	CCGCTCGAGCTAGCAGTGGCCGGAGGAGG

Enzyme sites were underlined.

Supplement 3. Primers used for site-directed mutagenesis.

Locus	Sequences (5'-3')
−719C>A	F: GGAGACCTAGTGAATAATGGTG
	R: CACCATTATTCACTAGGTCTCC
−249C>T	F: TCGTCACAGCGTGGTGGCATT
	R: AATGCCACCACGCTGTGACGA
−812A>G	F: GCTTGGAAAGTATGCCTATGTAGG
	R: TAGGCATACTTTCCAAGCATTCTGC

The mutated nucleotides were underlined.

Supplement 4. Primers used for real-time quantitative PCR.

Names	Sequences (5'-3')
CEBPα	F: TAGACAAGAACAGCAACGAG
CEBPα	R: ACCTTCTGTTGAGTCTCCACG
CEBPβ	F: CTCGCAGGTCAAGAGTAAG
CEBPβ	R: AACAAGTTCCGCAGGGTG

Supplement 5. Oligonucleotides used for EMSA.

Names	Sequences (5’-3’)
Bio-C/EBPa1-F	bio-CATAATGGTGCCAATAACCTGAGTG-bio
C/EBPa1-F	CATAATGGTGCCAATAACCTGAGTG
C/EBPa1-R	CACTCAGGTTATTGGCACCATTATG
C/EBPa1-M-F	CATAATGGTGATGCGGCCCTGAGTG
C/EBPa1-M-R	CACTCAGGGCCGCATCACCATTATG
Bio-C/EBPα2-F	bio-CGTTTGGGGGCAAAGTTCACCTGTC
Bio-C/EBPα2-R	bio-GACAGGTGAACTTTGCCCCCAAACG
C/EBPα2-F	CGTTTGGGGGCAAAGTTCACCTGTC
C/EBPα2-R	GACAGGTGAACTTTGCCCCCAAACG
C/EBPα2-M-F	CGTTTGGGGGGCCGGTTCACCTGTC
C/EBPα2-M-R	GACAGGTGAACCGGCCCCCCAAACG
Bio-C/EBPβ1-F	bio-GTGCAGATACACCCGCTGGAATGAA-bio
C/EBPβ1-F	GTGCAGATACACCCGCTGGAATGAA
C/EBPβ1-R	TTCATTCCAGCGGGTGTATCTGCAC
C/EBPβ1-M-F	GTGCAGCGCAGTTTGCTGGAATGAA
C/EBPβ1-M-R	TTCATTCCAGCAAACTGCGCTGCAC
Bio-C/EBPβ2-F	bio-ATTTGATTGCCAAGTAATATTCTCC-bio
C/EBPβ2-F	ATTTGATTGCCAAGTAATATTCTCC
C/EBPβ2-R	GGAGAATATTACTTGGCAATCAAAT
C/EBPβ2-M-F	ATTTGACGTATCCATAATATTCTCC
C/EBPβ2-M-R	GGAGAATATTATGGATACGTCAAAT

Supplement 6. Primers used for ChIP-qPCR.

Names	Sequences (5’-3’)
CEBPα-1	F: CCTGCTTTCTGGTTTGGG
CEBPα-1	R: GTAGGTGGGATGTTGCT
CEBPα-2	F: GATGGCGGCACCCTAA
CEBPα-2	R: GAGTGGCCCAGTCTCAAA
CEBPβ	F: GATTGACAGACCGATTTCCC
CEBPβ	R: CACGCCGAGGATTCATTAG
