# Supplementary material for: Transcriptional regulation of porcine PABPN1 gene in adipogenesis
Source: Anim Biosci. 2025 Jun 24;38(12):2584–96. doi: 10.5713/ab.25.0035 (PMC12580747; doi:10.5713/ab.25.0035)
Supplement: Supplementary file 2 [file ab-25-0035-Supplementary-7.pdf]

Supplement 7. Overview of RNA-seq data.

quality control

Sample	Raw Reads	Clean Reads	Error Rate	Q20	Q30	GC Content
NC_1	54338526	54005824	0.02%	98.20%	94.87%	48.40%
NC_2	42454738	42016980	0.03%	97.72%	93.76%	49.84%
NC_3	39251874	38906784	0.02%	98.23%	94.97%	49.51%
siPABPN1_1	44564418	44159326	0.02%	98.14%	94.77%	49.99%
siPABPN1_2	40862656	40527286	0.03%	97.73%	93.76%	48.63%
siPABPN1_3	41500282	41105084	0.03%	97.94%	94.26%	49.26%

map_statistics

Sample name	NC_1	NC_2	NC_3	siPABPN1_1	siPABPN1_2	siPABPN1_3
Total reads	54005824	42016980	38906784	44159326	40527286	41105084
Total mapped	51906164 (96.11%)	39246118 (93.41%)	36650978 (94.2%)	42065874 (95.26%)	37466648 (92.45%)	38359658 (93.32%)
Multiple mapped	1106632 (2.05%)	1519904 (3.62%)	1231646 (3.17%)	1926796 (4.36%)	951660 (2.35%)	1409324 (3.43%)
Uniquely mapped	50799532 (94.06%)	37726214 (89.79%)	35419332 (91.04%)	40139078 (90.9%)	36514988 (90.1%)	36950334 (89.89%)
Read-1	25399766 (47.03%)	18863107 (44.89%)	17709666 (45.52%)	20069539 (45.45%)	18257494 (45.05%)	18475167 (44.95%)
Read-2	25399766 (47.03%)	18863107 (44.89%)	17709666 (45.52%)	20069539 (45.45%)	18257494 (45.05%)	18475167 (44.95%)
Reads map to '+'	25399766 (47.03%)	18863107 (44.89%)	17709666 (45.52%)	20069539 (45.45%)	18257494 (45.05%)	18475167 (44.95%)
Reads map to '-'	25399766 (47.03%)	18863107 (44.89%)	17709666 (45.52%)	20069539 (45.45%)	18257494 (45.05%)	18475167 (44.95%)
Non-splice reads	28437685 (52.66%)	20880370 (49.7%)	19579548 (50.32%)	22433398 (50.8%)	20732810 (51.16%)	20816864 (50.64%)
Splice reads	22361847 (41.41%)	16845844 (40.09%)	15839784 (40.71%)	17705680 (40.09%)	15782178 (38.94%)	16133470 (39.25%)
