# Supplementary material for: Transcriptional regulation of porcine PABPN1 gene in adipogenesis
Source: Anim Biosci. 2025 Jun 24;38(12):2584–96. doi: 10.5713/ab.25.0035 (PMC12580747; doi:10.5713/ab.25.0035)
Supplement: Supplementary file 3 [file ab-25-0035-Supplementary-8.pdf]

Supplement 8. Differentially expressed genes.

Gene_id	NC_1	NC_2	NC_3	siPABPN1_1	siPABPN1_2	siPABPN1_3	readcount_siPABPN1
ENSSSCG00000031421	0	0	0	1.216545614	0.827038548	0.822424226	6.109797521
ENSSSCG00000028894	0	0.781806011	0	5.202633353	1.616860361	2.411759043	4.01844514
ENSSSCG00000004308	0.14295999	0.128560084	0.27481785	0.611085991	0.598221716	0.660982266	9.936078637
ENSSSCG00000009374	0.104822961	0.094264478	0.503763692	0.716909322	0.779797009	0.581584698	15.05349937
Novel00251	0.416218043	0.336864349	0.360050854	0.800610416	1.219176574	1.15464227	19.32881506
ENSSSCG00000000699	0.167995253	0.396568266	0.484416246	1.131007251	1.054474149	0.640805543	17.01729072
Novel00026	1.243924875	1.283132432	1.336285531	4.347582692	2.551594468	3.450807257	107.2839804
ENSSSCG00000016254	1.906013261	2.308688891	4.206130995	11.37296649	3.472451782	7.121973011	142.581499
ENSSSCG00000014015	0.741471588	0.733464061	0.142536124	1.140997872	1.378985383	1.64554988	21.35026723
Novel00082	0.542300467	1.20177365	1.005254766	2.632672803	1.404790957	2.578990455	43.20013237
ENSSSCG00000009699	0.271945396	0.524042571	0.504101364	0.946556071	1.219249539	0.943014288	40.52881054
ENSSSCG00000019224	10.65180715	5.91636981	4.516854186	16.06990997	18.35355005	17.38204715	20.93618276
ENSSSCG00000022347	0.468125235	0.330764081	0.932035489	2.029598726	1.274832208	0.711159718	45.4836166
ENSSSCG00000013387	0.983039363	1.105026163	1.299194102	2.626266205	2.742377998	2.499820916	24.3134293
ENSSSCG00000018868	8.33558471	11.94669859	2.253351976	9.353048725	23.98040086	18.78823524	25.38100352
ENSSSCG00000037312	0.779459245	0.660892689	0.417407659	1.313688125	1.522116382	1.420953134	48.40149296
ENSSSCG00000007490	0.385617847	0.769842246	1.312081529	2.334039485	1.204846044	1.583235033	83.91226098
Novel00423	0.381157509	0.635122871	0.387907787	0.747546102	1.376051371	0.808584592	33.14011279
Novel00182	5.673147077	4.554466853	6.650981735	18.77907091	6.927445747	9.312126362	450.2530009
ENSSSCG00000044226	0.287396839	0.34791113	0.201865766	0.529193475	0.565335791	0.643953463	59.74403285
ENSSSCG00000010026	0.836153275	1.141322643	0.731928197	1.697724924	1.818881128	1.974418452	139.686942
Novel00062	6.840067925	7.285511877	10.53532116	22.79129069	12.61685473	13.89442802	666.5528145
ENSSSCG00000008959	34.33037704	38.10694061	45.61982435	105.0206605	61.8474329	67.81324266	1433.888717
ENSSSCG00000035417	1.98591037	3.494105076	5.228447974	12.06889142	4.335714935	4.551053687	61.22013549
ENSSSCG00000032405	0.379972874	0.377667773	0.475745436	1.064280645	0.641583581	0.69348258	60.64369518
ENSSSCG00000013333	0.6701311	1.119171715	0.644110246	1.964222994	0.979240962	1.770504459	37.3506506
ENSSSCG00000008953	49.44328182	46.34828822	51.42264766	121.0470923	75.04206867	90.48462973	2660.999204
ENSSSCG00000015403	0.215249457	0.237560805	0.394974546	0.52696012	0.504949005	0.583558494	41.80071682
ENSSSCG00000008957	358.7286102	350.6929568	448.9263492	911.6739854	589.1213275	658.2805541	18246.98051
ENSSSCG00000040725	132.1450664	168.9222187	201.9967304	411.9990127	236.8161476	283.5156741	6098.477275
ENSSSCG00000009642	7.299771073	5.864397463	11.25332952	19.86277315	10.46411154	14.52875341	560.649236
ENSSSCG00000002383	0.587145345	0.384002952	0.718259545	1.323331803	0.670072598	1.061198658	43.39754185
ENSSSCG00000033815	4.929349097	6.025880215	7.329008586	14.02513405	9.239202064	9.294486675	106.8559276
ENSSSCG00000020970	23.42584858	17.27169764	26.57195391	52.24401693	29.96942008	38.07686522	626.9153702
ENSSSCG00000025005	0.478309012	0.526690365	0.520721969	1.151620496	0.748861641	0.798842293	69.9053736
ENSSSCG00000008090	51.82966881	39.62261368	54.22364445	112.0526564	66.58899155	78.55470937	2368.118122

ENSSSCG00000030655	0.8906476	0.514887105	0.749056176	1.468450955	1.257106158	1.103022658	91.33603195
ENSSSCG00000014310	26.82775455	29.57849953	40.31277444	68.80553791	40.880723	59.6408577	1398.154828
Novel00007	0.588377307	0.552116796	0.663884113	1.427008005	0.731489929	0.975792164	61.92084692
ENSSSCG00000021259	4.550002285	5.260750726	4.123423522	9.557841781	7.374092114	7.272843468	141.2679467
ENSSSCG00000004890	17.9872992	15.71399796	22.38179988	44.22382308	24.66002853	28.04598599	954.5378469
ENSSSCG00000047596	1.576152228	1.375703438	2.085285532	3.091232432	2.909766697	2.597748859	234.6762208
ENSSSCG00000022842	2.207937617	3.047571603	4.367792996	4.609198984	6.660820092	4.843104168	79.48695196
ENSSSCG00000038504	1.129274684	2.016118652	2.202775308	3.322189124	2.501732364	3.086683015	67.87348229
ENSSSCG00000015579	24.76559107	23.82325518	28.9975459	58.96144038	36.79480444	35.39241756	2649.717074
ENSSSCG00000006035	3.658909883	3.027683755	3.546389494	6.978901883	5.382386613	4.925873906	284.3511615
ENSSSCG00000015850	0.675977064	0.79185416	0.961770218	1.859152638	1.01732246	1.184366612	115.3645114
ENSSSCG00000051187	2.140749588	2.493903749	2.618795907	4.221794116	4.433782872	3.486745031	189.4790955
ENSSSCG00000008664	0.713595082	1.375107677	1.714716356	3.028492011	1.730022812	1.531836695	93.51634299
ENSSSCG00000004367	1.636108234	1.082660787	1.691264177	2.098075463	1.851531173	3.425489985	60.5232638
ENSSSCG00000031023	20.21803341	16.58156004	24.87421096	40.65313504	30.081123	31.7828711	480.8903445
ENSSSCG00000016285	2.299060933	2.748391676	2.223021823	4.872503581	3.533281549	3.418090804	217.1608022
ENSSSCG00000023522	3.198463388	3.406918676	3.943544625	7.099964505	4.522901486	5.415557888	390.5902588
ENSSSCG00000049380	2.342673967	3.242135162	3.114376889	4.408680682	4.583226221	4.979659965	116.2767755
ENSSSCG00000032225	0.390121729	0.467768009	0.708283274	0.917171708	0.624776207	0.961997998	87.69670158
ENSSSCG00000016218	0.579937245	0.648722498	0.815734496	1.469234905	0.848383106	0.961368258	58.58379594
ENSSSCG00000005385	7.663688682	8.447951205	9.682872368	17.06618727	11.2452255	12.97320622	760.0452482
ENSSSCG00000015413	5.591702912	5.144653017	6.109734998	10.12626427	8.448329662	8.329511093	658.4958294
ENSSSCG00000045652	6.115510665	7.649324996	7.374278969	11.97728542	11.01171085	10.33335609	75.83180129
ENSSSCG00000039890	2.516683263	2.416622075	3.751440625	5.03238027	3.569884882	4.910788115	80.14898967
ENSSSCG00000038888	6.106832335	12.05125363	8.15237097	14.71964558	11.43632938	14.66663223	182.6843159
ENSSSCG00000009477	3.632696039	2.956648186	4.861777597	7.626433968	4.85325358	5.357692815	294.1805182
ENSSSCG00000039318	0.639540551	0.67615655	0.67285552	1.329923584	0.976445027	0.791182836	90.59867369
ENSSSCG00000039651	5.106368735	5.144225423	5.032346467	9.338768313	6.461430811	7.979425828	279.1446394
ENSSSCG00000009973	1.142394003	2.152488977	2.091496033	2.953168047	3.111049267	2.213372919	115.5097322
ENSSSCG00000029037	1.546374519	1.489942635	1.62788477	2.801387694	2.208311134	2.230036595	149.2087808
ENSSSCG00000024018	11.99174366	16.6523629	16.62995979	27.42401086	19.10373784	23.20592893	848.6331802
ENSSSCG00000006862	5.53745336	6.395141192	6.725956123	10.16512178	9.760678499	8.285797937	376.4229781
ENSSSCG00000013307	2.397337671	2.425344146	2.749389505	4.366823362	3.457915226	3.627557667	106.3405933
ENSSSCG00000028741	2.293135209	2.821299952	3.269809661	4.71791596	3.304018999	4.543893848	126.1550702
ENSSSCG00000017391	0.516141261	1.070128203	0.757930181	1.041844297	1.213228554	1.232975144	92.42287168
ENSSSCG00000010948	107.5182301	100.4645309	131.3023945	193.9324821	140.575331	174.2938317	4413.680194
ENSSSCG00000023993	1.798866039	1.469110265	2.205378792	2.918794416	2.440865998	2.834617824	169.4513818

ENSSSCG00000048443	0.897919204	1.232094948	1.584744701	1.905853763	1.641153756	1.954101929	89.93309984
ENSSSCG00000009482	7.407960088	6.53135339	9.715366444	13.82027724	10.36409048	11.14190895	400.4321529
ENSSSCG00000025308	1.680967611	2.683177124	2.423544723	2.658574834	3.243490271	4.158037745	90.99023492
ENSSSCG00000008055	3.151417567	4.880752439	3.996662672	6.885081629	5.942394279	5.018806315	154.6966131
ENSSSCG00000042281	0.600113834	0.866306388	1.062547897	1.431112328	1.292313459	1.007637747	89.63955029
ENSSSCG00000032279	2.034508064	2.388616414	2.643558647	4.058392215	3.503489407	2.909091803	210.8851197
ENSSSCG00000008963	10.31832703	10.1156254	11.10995995	19.35359121	12.42629609	14.80750316	626.7216326
ENSSSCG00000015871	5.42074037	4.805087464	5.638241033	9.309812144	6.291915894	7.814300697	305.8935536
ENSSSCG00000001076	0.830472723	0.876221668	0.84169363	1.381288413	1.123974573	1.243160067	115.3402075
ENSSSCG00000001097	1.087653998	1.012331499	1.03496673	1.757399021	1.41091263	1.448300058	107.3714172
ENSSSCG00000038374	2.078447065	2.102728157	2.172544434	3.131741514	2.923273869	3.267331389	136.2414919
ENSSSCG00000005925	6.73704042	9.719422044	8.570441276	14.36802285	9.547893353	12.59286773	256.3040746
ENSSSCG00000000359	6.585257478	7.208269621	7.937884842	12.66698026	6.926712607	12.1289861	148.6338945
ENSSSCG00000005688	39.03044923	46.42190357	47.18085059	71.72036531	54.49897324	67.51496128	1189.278054
ENSSSCG00000050281	0.900625592	0.740088744	1.029830653	1.34077369	1.285113393	1.277943319	95.4453913
ENSSSCG00000012548	2.453654044	2.187565308	2.474780613	3.592097523	3.569357872	3.242701234	250.0477485
ENSSSCG00000027621	4.577269317	4.99601731	3.87898043	6.989865889	6.287113386	6.397431673	142.4892896
ENSSSCG00000040798	2.514028783	2.736080185	2.718461595	4.139759309	2.869105262	4.596657202	102.8026163
ENSSSCG00000006392	2.959141744	4.817466615	5.619825446	7.537014113	5.323114217	6.42569604	239.0229907
ENSSSCG00000017723	168.9115912	146.1843334	153.3397608	228.8257786	208.934999	243.0188822	2820.88142
ENSSSCG00000008988	3.798223072	3.805391595	3.840093708	5.537618874	5.517769553	5.552565573	266.7505918
ENSSSCG00000037821	2.561624459	2.239932823	3.117908453	3.928695925	3.71671399	3.749542098	224.0430634
ENSSSCG00000006172	1.631776855	1.489840869	1.715668788	2.640779888	2.305895828	2.016285414	247.187533
ENSSSCG00000012027	9.479412167	9.778779943	11.24768388	17.91310314	13.02727491	12.76062985	1501.397907
ENSSSCG00000001342	2.015160758	1.674648498	2.153086411	3.160976858	2.484581696	2.720287007	117.6237239
ENSSSCG00000031321	6.949817061	8.022110892	9.895312623	14.1884962	9.283985668	11.7980159	515.9329286
ENSSSCG00000002980	11.35702325	8.356145215	11.6377571	16.36918765	12.830147	15.49254122	120.476842
ENSSSCG00000031132	7.488778101	16.54957053	13.64555619	19.410905	14.97957228	18.74155022	421.8663725
ENSSSCG00000035037	6.08139038	8.569086353	9.489763301	14.339638	9.80664912	9.94002827	826.131322
ENSSSCG00000036169	6.371652843	7.201035864	7.075985499	8.943643488	10.32869014	9.912770066	171.6686699
ENSSSCG00000015398	1.06720589	1.408935416	1.636861948	1.819866151	2.169538389	1.779489205	128.6554043
ENSSSCG00000018055	7.344132923	12.66661275	11.43130725	16.30545002	13.45472452	14.19054441	304.3265045
ENSSSCG00000023850	4.880704205	4.474810719	5.112662913	6.943364061	5.85048157	7.563191734	135.1320254
ENSSSCG00000015334	1.438692798	1.451679863	1.584264811	2.120929725	1.88034579	2.278394405	140.3158494
ENSSSCG00000024388	40.06061078	45.74359322	49.83527812	73.24958794	51.02417122	65.8016425	1837.468681
ENSSSCG00000036213	17.51086271	16.9065398	17.64443093	26.25159722	24.43501257	22.37037335	2566.400627
ENSSSCG00000009782	4.148220518	4.602421132	5.048660411	7.322949679	5.034685245	6.89963599	271.2789831

ENSSSCG00000045632	1.352455711	1.459472572	1.16994648	1.821048135	2.0279532	1.719614291	125.0806965
ENSSSCG00000014892	1.370390883	1.237207557	1.602395281	2.11709938	1.821178531	1.915787184	137.275761
ENSSSCG00000016900	7.402927457	7.129160712	8.241341874	10.52663545	11.05791322	10.06036917	427.4156284
ENSSSCG00000031011	8.215918809	7.490971456	6.416230278	9.657765418	10.08050758	11.18496947	205.8604974
ENSSSCG00000043173	18.88167341	18.22930271	18.49610997	27.72785123	25.06266307	24.55321193	514.1145525
ENSSSCG00000010084	3.128416272	4.631653821	4.510412156	6.523166966	4.328183523	6.067983987	168.2684664
ENSSSCG00000023351	29.06817315	24.27404223	32.2344908	46.96865617	35.11560824	36.7686231	1984.414826
ENSSSCG00000023176	3.301652845	3.35636017	3.357419513	5.338394241	4.18259027	4.358367448	220.11008
ENSSSCG00000012026	7.111010352	6.37609731	8.204861085	12.61486136	9.268119754	8.166803107	732.8875216
ENSSSCG00000014924	35.55949396	31.67602269	38.99054718	57.35405246	44.31608363	45.15694877	3597.76574
ENSSSCG00000006864	2.570280025	1.821683908	2.072688617	3.053937166	3.180044807	2.759461228	156.7566159
ENSSSCG00000025631	7.855004829	8.170973069	8.733384345	11.58859838	10.78479349	11.61264754	349.3438605
ENSSSCG00000040961	14.95931516	15.46992584	19.48048397	28.93936001	19.86346608	19.65295234	1685.572107
ENSSSCG00000023886	19.54832573	25.00689609	23.52541337	34.39072719	26.14018145	31.82065357	578.4900588
ENSSSCG00000046151	3.636274564	2.938483018	4.421195474	5.823382265	3.926744354	5.183204592	225.4074057
ENSSSCG00000006243	15.14894334	16.59580769	19.18864949	26.61448078	17.68221746	24.68078276	606.977256
ENSSSCG00000032668	1.61005259	2.371851315	2.127860702	3.422993616	2.531423167	2.282220964	294.9004308
ENSSSCG00000008645	52.63245886	49.49090302	54.85468473	77.53731561	62.19814182	72.76318072	1544.598925
ENSSSCG00000014221	23.36499704	19.43412003	25.97699911	34.63569259	29.1699979	29.30645497	2074.893748
ENSSSCG00000046793	23.06625737	24.79667549	26.16581693	34.8343705	31.84724954	33.13123533	215.7405602
ENSSSCG00000007477	1.199024946	1.253022421	1.006825923	1.824818421	1.534615972	1.325016808	179.843405
ENSSSCG00000030165	9.112779494	8.487552385	11.63687057	15.38635601	10.62270448	13.33220098	458.6123678
ENSSSCG00000048367	3.328353816	3.586071814	3.500918856	4.268139068	4.671740998	5.081207915	169.6244874
ENSSSCG00000013875	2.879417193	3.33957762	3.414248922	4.279091523	3.716057475	4.927099147	243.1237629
ENSSSCG00000025588	4.744153728	5.999470585	6.09179567	8.365088228	6.539818887	7.634345171	307.7107254
ENSSSCG00000032242	7.106577345	9.183916267	9.099548905	15.23110545	9.358034245	9.443686712	346.1285557
ENSSSCG00000031775	4.377029566	3.987417716	3.882477995	5.286714612	5.64035283	5.584549883	476.5911586
ENSSSCG00000006240	3.160010159	4.235759955	4.670578242	5.352044598	4.740387857	6.009583898	205.0969119
ENSSSCG00000013913	4.706206612	5.778748231	5.979569795	8.375805781	6.737639855	6.923957401	448.9402446
ENSSSCG00000034044	6.264222763	8.218367934	9.608834038	12.36124043	8.617935546	11.14874425	284.1188145
ENSSSCG00000042185	2.611547124	2.847725098	3.021188945	4.010702657	4.100772316	3.188565075	182.8538601
ENSSSCG00000032188	3.366165091	4.801840965	3.822422315	4.8132297	5.027692996	6.115264389	271.3090179
ENSSSCG00000022998	11.05618762	13.63911573	14.98662761	20.43891674	14.27933743	18.00081024	423.3408302
ENSSSCG00000007556	14.45195531	19.16947725	17.10303905	22.70240009	18.56239687	26.1430504	283.3624817
ENSSSCG00000037116	1.861864722	1.730604835	1.89483824	2.427703288	2.509720824	2.38720877	237.0157531
ENSSSCG00000039953	6.568851895	6.784942145	6.964339527	8.47843314	9.699174631	8.894009991	481.0622067
ENSSSCG00000009164	5.61161651	7.340183572	6.496981217	6.941690328	7.906407634	10.96790176	230.8030216

ENSSSCG00000007436	5.230604889	6.979130392	8.154364824	9.670440473	8.165861561	9.123050612	481.2627438
ENSSSCG00000030857	23.54227727	30.81290224	26.9892348	38.86411077	35.97016857	33.20357427	2970.374461
ENSSSCG00000037241	22.47501817	23.03454869	23.51598866	33.33809647	26.54267876	31.8646942	606.3100959
ENSSSCG00000049515	6.067739672	8.069010184	7.799101561	10.9007556	7.305813484	10.79832855	256.3792524
ENSSSCG00000034313	77.23062402	79.15935058	91.5480144	131.7168331	88.27317025	108.4002843	1173.168754
ENSSSCG00000030484	10.64252109	9.408321314	10.69987363	13.77969475	14.11383109	12.86748063	1201.131552
ENSSSCG00000001513	2.079671131	2.132433475	2.113247834	2.952267472	3.018567506	2.373705248	275.1631069
ENSSSCG00000035392	66.66231356	90.75771958	96.01668616	128.7922426	84.88232438	118.8511235	2577.75019
ENSSSCG00000004962	7.729754541	6.642456991	8.464979119	11.35437397	8.767453171	9.900711106	641.6095058
ENSSSCG00000012277	1282.412534	1414.443819	1505.304716	2077.835406	1589.256398	1844.605079	24448.62941
ENSSSCG00000029160	3.261067783	3.578458994	3.507590033	5.34348053	3.573969874	4.631008153	264.7952449
ENSSSCG00000017605	13.42739074	14.33810257	13.83091903	18.091989	17.51082486	18.89159914	931.8213647
ENSSSCG00000045477	2.915195682	2.559408295	2.68122423	3.915570747	3.137322544	3.660122124	215.7195266
ENSSSCG00000022401	10.14592905	13.43609797	13.50044212	16.62625839	13.3769539	18.32637142	594.3858829
ENSSSCG00000005832	2.72320332	5.056953663	4.1240659	5.539224615	4.63471423	5.300183968	542.0899641
ENSSSCG00000024827	4.446959265	3.934826556	4.646815587	6.67046197	5.235412344	5.121318512	211.1157112
ENSSSCG00000027607	30.09650127	29.66911474	32.53178619	48.16795975	34.22840851	38.12378674	910.4230083
ENSSSCG00000011470	3.645180027	5.141796943	4.654835356	5.555886214	5.636494341	6.240670299	211.9056402
ENSSSCG00000010007	13.03629767	15.52699475	18.52855919	20.27404195	17.86122911	22.95539348	334.5367212
ENSSSCG00000034758	5.591043402	6.876674481	7.097736633	9.709202552	6.778056128	8.9355052	808.6199432
ENSSSCG00000022742	164.0692242	165.5410479	182.919743	245.7982503	186.7075393	234.0906996	5241.566978
ENSSSCG00000033374	37.21457206	46.35649757	52.51831976	61.63493182	48.01969595	66.4993953	735.5490114
ENSSSCG00000040631	58.92464846	62.99079243	64.4209186	87.9633408	75.20524677	78.14422639	4059.814608
ENSSSCG00000015271	7.60246628	7.985086873	8.63794591	11.86112403	11.07039527	8.293719642	661.817242
ENSSSCG00000001765	4.703744045	6.66560847	5.630972953	8.361885401	6.667587499	6.80704011	650.5654148
ENSSSCG00000013299	17.32491871	16.95320161	17.18160603	21.70305552	19.27660733	25.35038302	335.3666386
ENSSSCG00000008038	4.903572297	6.502839821	5.84671603	7.959660896	6.349116992	7.777322035	270.1137274
ENSSSCG00000022129	8.927181955	9.215787945	10.37545366	12.17792932	11.56261634	12.82480905	406.4444564
ENSSSCG00000011133	1.93572613	2.284729894	2.26756098	3.258009145	2.700040786	2.33886613	277.5146001
ENSSSCG00000012997	13.55309825	17.64564533	16.91070997	21.91643639	19.62821327	19.93306445	989.4509917
ENSSSCG00000033920	5.115032608	6.977460729	8.247118849	9.903618848	7.637127871	8.353969586	454.6097304
ENSSSCG00000014362	4.453577476	4.383231138	3.828801193	5.859142913	5.314759778	5.033435213	248.6220022
ENSSSCG00000027060	13.37305843	18.01627253	18.2825677	26.09165827	19.35993033	17.75298232	946.437021
Novel00100	18.21174729	25.9072511	20.06992275	27.38051478	25.25659977	28.96288512	394.6965548
ENSSSCG00000027169	6.877725676	7.974421309	8.216910574	9.290416702	9.189156386	10.85291569	384.4321123
ENSSSCG00000006151	5.574219692	4.88438752	5.458865642	6.088444179	7.460820331	6.752300106	342.1982399
ENSSSCG00000040349	12.71660273	12.44731933	14.12676323	16.20269579	16.98726709	16.68896553	774.5442278

ENSSSCG00000037016	150.6469868	198.8222236	194.9990479	259.9881863	198.9341706	230.5190539	5845.561575
ENSSSCG00000033830	12.01735537	13.78098731	13.92696707	18.26590402	15.43781927	16.53258553	388.1865054
ENSSSCG00000009031	8.030985939	7.794219712	8.534549613	11.66448741	9.164071329	10.05430754	828.8311694
ENSSSCG00000044913	10.44711659	11.1522328	12.25339036	18.17486561	11.76979233	12.90324866	889.62107
ENSSSCG00000021991	4.173983321	5.996858086	5.539047038	7.094775001	5.779953367	6.941217625	664.3471562
ENSSSCG00000035774	25.3195963	31.0595041	27.49219474	43.72068951	31.67726422	30.70505843	935.5941629
ENSSSCG00000012202	10.40302173	8.72441085	9.450202236	12.16236185	12.45004123	11.65737347	739.548587
ENSSSCG00000017472	109.0337332	140.1942144	158.3106818	196.5443244	148.0830742	168.9683147	11636.57786
ENSSSCG00000003262	15.38107867	18.13538762	17.45065754	23.73570879	17.86987837	22.52266544	435.6566367
ENSSSCG00000033497	16.28661656	20.69778011	16.55528256	24.39429896	22.64664742	20.38353726	1683.339127
ENSSSCG00000038633	5.000505023	5.874877792	5.736596874	7.216861772	6.549940698	7.085183117	294.4516872
ENSSSCG00000008835	53.05467321	60.45742067	63.64728892	79.07554676	64.91807478	78.67855477	2258.032633
ENSSSCG00000001341	35.98784305	38.39128565	46.247775	56.29155971	42.77451761	52.32359926	1302.691117
ENSSSCG00000004651	97.6580727	83.99970071	90.63419903	114.0201557	111.3059945	117.3018245	6745.302345
ENSSSCG00000041401	6.289431546	6.748537433	6.981194131	8.134037282	8.523564447	8.451225007	355.7700093
ENSSSCG00000022725	17.43022988	20.20786052	20.20265691	24.25095505	23.47824259	24.49288462	641.8859916
ENSSSCG00000012173	63.27830893	58.0577117	66.73388544	78.88422963	76.91615754	79.65544703	1487.238836
ENSSSCG00000011106	12.05050952	14.10508344	11.35047817	17.03243062	13.94542061	15.944407	491.6390628
ENSSSCG00000017297	9.167835125	12.48926667	11.14594462	12.92275459	13.49815162	14.45730803	569.0105138
ENSSSCG00000016929	8.954179129	8.720718907	10.95312227	13.10032108	11.7287179	10.92545647	1326.512151
ENSSSCG00000022820	19.85941711	26.1453335	23.17285556	28.45239293	23.67091394	34.1216	778.3807396
Novel00168	6.355674534	6.467525475	6.537580696	7.911934833	7.776482699	8.480627655	329.2446409
ENSSSCG00000039612	69.04051086	83.04899014	84.96980351	105.7405962	89.78609185	99.53291287	879.217623
ENSSSCG00000007165	22.07937617	28.1152333	30.55974489	35.95833664	29.19587852	35.15808738	574.8316378
ENSSSCG00000003805	7.106757513	6.12217879	8.18619551	9.67625435	8.28457756	8.730583926	723.1966043
ENSSSCG00000037452	4.100091768	3.823365035	3.778111483	5.086333481	4.886014009	4.648578239	415.1434949
ENSSSCG00000022246	28.69659816	37.58892183	37.38247401	50.73214613	34.97566592	43.22768115	1413.662964
ENSSSCG00000024108	4.005956712	4.436700863	5.046060254	6.859724074	5.285202895	4.629039919	704.0805602
ENSSSCG00000022757	4.361169404	5.33934213	6.279891212	7.359022417	5.581927503	6.879951425	921.0355588
ENSSSCG00000013408	57.13849441	68.7717061	68.19515977	88.7027555	69.28396194	83.20874868	2419.095481
ENSSSCG00000010551	6.703617824	7.460124825	6.738638526	8.333764958	8.857018203	8.807601965	353.4864929
ENSSSCG00000006559	36.12988827	39.06999388	35.07425266	45.32758112	45.02011187	46.85703283	576.8027211
ENSSSCG00000023837	159.9201589	204.4946337	212.5440555	259.4742161	201.1063721	251.5652285	4612.977526
ENSSSCG00000016101	7.862711179	8.901833518	8.630298326	11.75016512	9.974916302	9.613007824	646.3199269
ENSSSCG00000038459	14.95212477	18.02992363	15.02479314	19.70657771	18.95992606	20.58243873	397.2229735
ENSSSCG00000004458	9.550489448	8.487457822	10.09761361	12.72742062	11.44078899	10.5977134	469.7227818
ENSSSCG00000031117	19.68996422	16.23110196	19.68853427	24.84231973	21.06594321	22.75936996	492.4008481

ENSSSCG00000011806	32.7209412	30.9239744	37.78208716	47.29622422	38.97835656	38.69768631	4163.254195
ENSSSCG00000032265	31.64533559	35.31525407	40.2784972	50.09115593	37.95684215	43.81676481	1196.413314
ENSSSCG00000032787	5.096827956	6.944747651	5.518723401	6.946105945	6.907814627	7.71362186	529.2451656
ENSSSCG00000037274	669.1393643	652.8532972	703.3387233	878.7945053	736.8887916	874.4411293	14080.31364
ENSSSCG00000029989	49.82669727	49.01473569	53.17314472	63.39709731	60.77008982	62.33635446	3642.789169
ENSSSCG00000013772	14.60348259	13.62186019	11.56728881	16.06552408	15.10952477	17.7985711	489.8641973
ENSSSCG00000023716	19.10832647	20.38151285	22.13645115	25.71714559	23.58764029	26.12342031	625.4539158
ENSSSCG00000023972	68.8287753	62.96144406	75.42845367	93.01978109	78.90278562	82.21418603	2496.22043
ENSSSCG00000039372	28.77141947	40.47223205	38.63054897	49.09566067	37.61348825	45.11489927	1163.809756
ENSSSCG00000035895	17.90682899	17.44172873	17.55318361	22.59258402	19.49219295	22.80404653	739.1849358
ENSSSCG00000031356	22.19044849	23.4701247	22.99511735	29.10281452	24.44525794	30.51725015	675.00316
ENSSSCG00000028304	52.17004349	53.82394688	52.80325571	68.09598296	65.57616536	60.78925732	3273.295041
ENSSSCG00000038762	23.28832006	23.3534251	23.58707234	27.44086856	28.5189163	29.99508053	1347.540017
ENSSSCG00000014249	16.05886777	19.75322497	18.83550636	22.49217136	20.33411327	23.82660459	512.6942194
ENSSSCG00000026454	14.42289762	13.11716098	12.70486796	15.78060387	16.68475057	16.87813554	725.87198
ENSSSCG00000008873	12.51366509	13.17448198	14.04461671	17.09069745	17.17082644	14.25270657	482.6972913
ENSSSCG00000034290	6.510771732	7.340549849	6.424751757	7.957492246	7.958438424	8.85755816	451.8164358
ENSSSCG00000033741	10.18131061	11.52727653	11.02125429	11.85748793	13.33997101	14.70090705	605.4336494
ENSSSCG00000002812	10.79586315	13.21456035	13.16354099	11.64491188	10.01610144	9.754852966	644.1936762
ENSSSCG00000012328	23.16949797	20.41077541	22.00531503	18.75118777	20.60368273	16.25365459	4065.959954
ENSSSCG00000032527	63.58055824	69.48261355	71.96464217	60.51784962	63.15574399	49.78443497	2338.456779
ENSSSCG00000025703	9.660733399	7.727654369	7.799914052	6.634048572	8.442237869	6.291326849	1120.10429
ENSSSCG00000024088	6.754438576	6.908599188	7.211484582	5.883748819	6.361915636	5.405390224	820.9154814
ENSSSCG00000006137	19.63249004	17.05414804	18.13368579	14.69091608	16.38366364	15.3590459	1258.171352
ENSSSCG00000001819	28.44540401	28.73941364	29.25481784	23.81647777	26.00071985	23.20917694	2441.752801
ENSSSCG00000004979	6.300332111	6.496586384	6.520865353	5.363617092	6.210511205	4.743777064	704.1653353
ENSSSCG00000011609	204.9679433	230.710742	213.5033889	171.8898669	190.2488439	185.529063	12145.58488
ENSSSCG00000017082	1967.727861	1827.788133	1801.54156	1521.066293	1564.273228	1639.714202	55909.64246
ENSSSCG00000023324	3.054639357	3.046565742	3.362444512	2.896254555	2.98888641	2.096600151	575.846882
ENSSSCG00000030108	13.68709021	12.54326127	12.98421853	11.02598905	11.85993726	10.20359595	1315.110933
ENSSSCG00000017000	10.54782325	10.50601205	8.662054347	7.477398037	9.280698592	8.333467879	610.301304
ENSSSCG00000040288	9.175106367	8.714083179	9.412228329	7.418118217	8.620717888	6.973533085	854.0522204
ENSSSCG00000016781	25.64379409	24.12706599	25.5724986	22.46543563	23.71528683	17.33081586	2798.977377
ENSSSCG00000011811	15.55218684	15.02835582	14.48192586	11.73334932	14.80180945	11.46770993	763.5461772
ENSSSCG00000006502	64.95907677	66.04300411	63.72617184	50.53859256	60.90428141	52.58385911	4179.821998
ENSSSCG00000039862	25.30701016	27.41600408	26.37864063	20.45964697	23.90636752	22.20574386	1238.344791
ENSSSCG00000005946	10.85241723	11.76378873	13.04959971	9.624258157	10.28007041	10.05615295	505.34586

ENSSSCG00000029760	19.00778118	20.40470494	19.72243205	16.35934592	17.50854314	15.88296563	983.2857547
ENSSSCG00000015567	36.92196113	33.70712191	35.26485505	25.9181802	35.22377664	27.97122035	2475.830848
ENSSSCG00000004123	15.37082887	13.6930351	13.73150087	10.64323177	14.21253351	11.15967452	2152.968191
ENSSSCG00000009593	10.6736414	10.69926705	10.44743127	8.3819768	9.424571482	8.947532667	553.439919
ENSSSCG00000006725	36.17099745	29.98488918	32.6982824	25.08483773	29.02163062	29.0405964	1776.154763
ENSSSCG00000009071	15.20219343	14.90130801	14.23199048	11.59281549	13.44379631	12.23012236	931.0966865
ENSSSCG00000016578	19.82748977	27.57545562	23.18994796	19.17300372	22.06353738	17.92217269	2920.964946
ENSSSCG00000016943	27.88944172	22.73183786	23.77324038	17.46225269	25.36575214	19.68693559	2007.304672
ENSSSCG00000017403	41.22837931	39.20887576	39.13632648	32.01990494	36.51586757	31.76011099	2708.184037
ENSSSCG00000009567	7.721488676	8.314646986	8.083991963	6.621313272	7.149472118	6.428738816	541.7216382
ENSSSCG00000038144	154.9601644	144.5583525	153.9073166	125.1542569	142.9427318	111.9223244	10384.13175
ENSSSCG00000004823	6.627457815	5.868116101	7.366405696	5.644033589	5.925596371	5.060856321	659.9044603
ENSSSCG00000005078	6.211657976	6.48158039	5.950725347	4.502848556	5.886001846	5.226546021	385.239533
ENSSSCG00000020749	7.623722231	8.710127915	8.954319298	8.027552121	6.623283407	6.449589502	324.8607905
ENSSSCG00000010548	19.26961147	15.71504623	18.18394804	13.57244948	15.77867094	15.11350965	432.9997043
ENSSSCG00000009833	8.213581148	8.479117207	8.727081195	7.177075477	7.923557504	6.096808023	576.1388384
ENSSSCG00000004509	176.5510372	154.5979801	160.7053473	130.5876785	147.3407006	132.4243866	8425.234272
ENSSSCG00000007438	14.1135249	14.90006875	14.07580489	12.61416375	12.26016253	11.04622462	616.3896539
ENSSSCG00000029874	8.313099818	9.783911454	8.324819622	7.201578556	6.983777609	7.82251426	704.3923123
ENSSSCG00000014207	22.92748146	16.8725639	18.99226949	15.58684361	17.71942087	15.6521488	2349.64299
ENSSSCG00000038071	16.30016596	17.86985167	15.87818071	13.45751239	13.66400064	14.44564797	527.4683124
ENSSSCG00000021232	22.79770326	24.65486499	24.07614331	17.06705255	20.14038825	22.093019	837.445762
ENSSSCG00000015814	10.97385319	11.53914201	13.62901407	9.972568059	11.06431466	8.869184701	438.0418632
ENSSSCG00000001641	13.56754641	10.30802092	12.85203322	9.808768633	10.78075938	9.903705524	1061.783859
ENSSSCG00000000145	242.295499	238.8786581	269.1516887	208.7293381	248.5650096	164.2069225	4525.948409
ENSSSCG00000006321	25.96534637	31.62084901	28.88772606	23.90043463	24.00374989	23.57461517	595.0954347
ENSSSCG00000024626	6.829152873	7.436743866	6.4473146	5.677163978	5.872201587	5.609051066	679.9146821
ENSSSCG00000016684	13.58368029	13.11012482	12.75022734	10.12713895	12.11334636	10.38977638	934.2957765
ENSSSCG00000032531	6.030263812	5.007918168	4.990869096	4.335470988	5.208120323	3.727981204	790.0259171
ENSSSCG00000009968	3.941797365	3.053108722	3.649486101	2.839377443	3.286720036	2.684471803	407.9978225
ENSSSCG00000024418	37.37223045	34.77141011	33.30699641	23.05211388	33.92296569	29.98744559	1754.066562
ENSSSCG00000001689	43.94994924	46.88883989	47.42471335	35.24095144	39.15991387	39.48339606	1992.754842
ENSSSCG00000038929	101.3687992	99.2741213	97.16427052	90.0126371	81.84506656	73.78374576	8456.60357
ENSSSCG00000015396	46.27675456	38.64547987	37.2138165	28.74262712	39.1112946	32.94684623	2555.190368
ENSSSCG00000022112	18.23264331	16.45408253	17.44302472	14.06452166	16.34430489	12.5230248	1855.008802
ENSSSCG00000009850	12.77792745	11.17585707	11.648825	8.779855526	11.12650079	9.431966602	795.4633147
ENSSSCG00000022478	13.36783733	13.20431378	13.20710317	10.94105035	11.48479724	10.31262922	778.007027

ENSSSCG00000016678	3.348510387	3.475219451	3.835872597	2.952678689	2.927728981	2.862708689	315.2516379
ENSSSCG00000011928	21.78610768	21.25800513	20.37818607	15.57214181	18.0255255	18.51552209	2200.923677
ENSSSCG00000011074	22.86068853	20.71026423	19.30776221	15.70652672	20.62237771	15.34947099	2048.252107
ENSSSCG00000022839	4.358605328	4.659770924	5.041992495	4.101729228	3.926150987	3.490159026	341.708751
ENSSSCG00000029039	2.91141774	2.567577193	2.737816819	2.210080495	2.585972317	1.959866303	380.0695095
ENSSSCG00000005785	19.49508555	19.79650697	17.76846309	15.0129838	17.31582161	14.52759512	923.113844
ENSSSCG00000037977	3.495357286	2.690765358	3.182811458	2.44699197	2.802524416	2.456664467	540.9005668
ENSSSCG00000013876	14.39323455	13.92434624	14.47862799	11.83859644	13.45506611	9.813123926	1458.231068
ENSSSCG00000005110	2.228604417	1.820689245	1.82712005	1.561113499	1.682921067	1.58624298	563.5384053
ENSSSCG00000004082	11.56735672	10.06484454	11.34857922	8.971363135	9.709883619	8.35479009	985.0957969
ENSSSCG00000011882	15.56341212	12.64542197	13.5397331	10.05871704	13.7530044	10.38522114	2088.001978
ENSSSCG00000008826	9.973548591	8.237689129	9.047953379	7.206200506	8.688193349	6.429407172	1205.854549
ENSSSCG00000012001	14.06141454	13.07307044	12.40594235	9.707929771	12.70278266	9.876801769	1090.898941
ENSSSCG00000025858	39.12254703	65.50133384	52.64280683	38.95198574	44.17428488	44.44904854	1547.528672
ENSSSCG00000004022	9.715953656	12.00164757	11.16751446	9.413712104	8.633293973	8.597604348	749.5054012
ENSSSCG00000010554	37.49864914	33.80719604	33.86172704	26.29774434	31.47955249	27.68823107	2499.028807
ENSSSCG00000031949	7.392236614	6.499070185	6.990718905	5.074778497	6.666802417	5.222680061	559.100896
ENSSSCG00000009157	5.840890581	5.647329047	5.982738576	4.07689484	5.994339788	4.102121553	388.447385
ENSSSCG00000009338	1.685733648	1.404469241	1.689594139	1.173336189	1.496307051	1.206621938	217.786666
ENSSSCG00000011497	4.200206278	3.306671457	3.340317054	2.73141845	3.523525481	2.560517874	298.6730531
ENSSSCG00000005724	8.225105056	7.06243188	7.508854439	5.683237574	6.803791403	5.986920254	849.7229087
ENSSSCG00000039408	22.80728896	23.81406472	23.43100782	18.85942372	19.26622736	18.41155814	1408.097999
ENSSSCG00000021562	3.167595197	4.039987268	3.747198848	2.655915929	3.497827976	2.647460477	219.5355826
ENSSSCG00000007135	13.24470579	13.92328095	14.30698879	11.38849053	11.34688867	10.6315144	826.3769437
ENSSSCG00000029967	3.727407474	3.809042683	3.517534583	2.969312232	3.2928021	2.647745209	199.6737931
ENSSSCG00000027667	10.82447395	10.38644808	10.1681943	7.770410619	9.728401692	7.785734767	573.7542275
ENSSSCG00000023027	2.871966616	3.033874071	3.093033881	2.366514105	2.59019421	2.271773028	158.6540558
ENSSSCG00000011760	16.71506364	14.80104197	13.75768902	11.44278212	13.31006596	11.75528962	433.0276283
ENSSSCG00000024678	3.466799581	5.291715574	3.713295777	2.924786853	3.572901661	3.494562318	360.636012
ENSSSCG00000011230	5.191081757	5.644171989	5.165467314	3.638615543	4.43200302	4.751877188	248.4126121
ENSSSCG00000037516	4.768802049	4.067011579	4.589404694	3.358028337	3.82014676	3.598894322	227.1793311
ENSSSCG00000005471	6.324733847	6.926266178	6.735780273	5.115027903	6.209235607	4.646227813	183.4759485
ENSSSCG00000001052	4.033937411	3.83444922	3.554193174	2.99486544	3.175314675	2.977631787	196.3367226
ENSSSCG00000010316	2.049721766	2.263196009	2.121069638	1.695790856	1.879131828	1.559116957	157.245846
ENSSSCG00000026886	2.482551644	2.423352828	2.447972907	1.830935461	2.200867812	1.831753369	346.2725652
ENSSSCG00000010513	3.159090451	2.911204249	3.346079731	2.390540034	2.80965481	2.299392503	303.5300867
ENSSSCG00000009473	16.42126984	14.40815266	15.06829527	12.00970042	13.59784465	10.95227033	2897.463762

ENSSSCG00000009943	5.394806388	4.636740755	4.983422156	4.505928628	4.301860763	3.152106821	316.8588895
ENSSSCG00000012490	7.340075126	7.098326371	6.903108473	5.184170177	6.142966789	5.670120301	190.5851489
ENSSSCG00000010034	2.407752855	2.165227559	2.364571042	1.67804328	2.0686078	1.778757375	160.3960302
ENSSSCG00000017277	4.698665273	4.225383647	3.591020926	2.998736425	3.322471678	3.635836605	231.8460009
ENSSSCG00000013614	110.6166057	120.8497984	109.7855548	83.67072221	89.51439934	96.97200066	2742.80745
ENSSSCG00000018057	2.236091799	2.453927645	2.305906858	2.012088286	1.850257075	1.661197444	184.3755913
ENSSSCG00000015862	19.55444651	20.11026428	16.8956466	12.92321972	16.02352143	15.77381787	490.0759444
ENSSSCG00000023437	3.713776922	3.736358689	4.206996547	2.436571235	3.321490683	3.414198538	376.7362073
ENSSSCG00000023174	12.42650932	11.6498563	10.39596166	7.342330599	10.78937411	9.007589541	930.7094379
ENSSSCG00000017268	9.409570833	9.252395015	9.91207988	8.023974521	8.042872176	6.394004158	358.7854706
ENSSSCG00000011243	2.660954074	2.33427451	3.084201561	2.286014012	2.280343195	1.761024357	184.1083012
ENSSSCG00000001716	11.40531119	11.48827482	9.833961316	6.739286199	9.202067007	9.745264298	349.2639031
ENSSSCG00000011776	11.12235016	8.41272536	9.252243618	6.909816278	8.721130594	6.96194716	728.3724246
ENSSSCG00000020736	3.880435321	3.651877045	3.825172428	2.823650199	2.995817563	3.079240817	124.8360975
ENSSSCG00000026516	2.906628052	3.209130726	2.583275988	2.706147999	2.096572903	2.002032696	172.8827083
ENSSSCG00000011046	68.31079023	60.42301116	60.41499025	43.31370122	56.85284168	47.68410112	4547.795393
ENSSSCG00000042150	47.75320486	42.43893314	37.48154727	27.92517865	36.34736221	35.63548981	1241.296212
ENSSSCG00000035595	4.116310093	3.657975596	3.824921171	3.06085501	3.497071626	2.473326054	893.5063662
ENSSSCG00000022895	2.824268029	2.147872846	2.471224907	1.82959351	2.411196926	1.560222191	301.6274924
ENSSSCG00000016611	2.826027937	2.581498033	2.759183554	2.187102375	1.839541728	2.324421318	162.109854
ENSSSCG00000016401	4.991597499	6.446313322	5.203447102	3.878109724	4.932165781	4.074913979	490.9512385
ENSSSCG00000028076	2.848239525	3.06765787	2.355647002	2.279248898	2.355996526	1.791592432	147.3149611
ENSSSCG00000027935	3.076076201	3.029478062	3.127297429	2.393854422	2.429808781	2.336375972	377.6775548
ENSSSCG00000009625	8.447761315	14.07142909	12.87163168	8.412026717	8.971399105	9.809040688	215.1885791
ENSSSCG00000010209	5.081809803	4.772400731	4.525877868	3.118115439	4.468348834	3.51547563	218.5991699
ENSSSCG00000009122	11.10188351	8.743829352	7.881050538	7.599047151	7.320995271	6.508520316	224.193303
ENSSSCG00000049158	3.082852853	2.575707951	3.71969545	2.46731972	2.937897795	1.78929624	249.8381584
ENSSSCG00000017694	10.26148056	9.005271995	9.78372441	7.618302013	8.181955149	6.499333399	1128.636828
ENSSSCG00000042787	2.543836128	2.32256697	2.930869161	2.008546246	2.386166705	1.556222094	135.5933386
ENSSSCG00000008765	13.9077474	12.42050485	12.65643674	8.794203412	10.96817555	10.02940732	667.8614254
ENSSSCG00000003741	1.891528146	1.661746667	1.776125341	1.256344217	1.461262921	1.358926992	106.4193395
ENSSSCG00000002263	4.849867885	4.741877102	3.261520685	3.25572202	3.473213552	3.115224035	153.1190536
ENSSSCG00000051565	2.936459148	3.102797762	3.104681704	2.397425665	2.416586734	2.145143501	179.9413649
ENSSSCG00000001532	3.666490809	3.236975575	3.637963856	2.760297952	2.815678639	2.457115684	197.4076902
ENSSSCG00000022987	3.302343654	4.233841983	3.367135832	2.556131333	2.842603394	2.868009898	140.7247598
ENSSSCG00000009125	12.45422519	10.50110978	10.68396306	7.908748612	9.135997366	8.554512225	813.5287518
ENSSSCG00000031175	3.439479107	3.293796012	3.560743673	2.630281081	2.73450353	2.44828985	283.4673908

ENSSSCG00000004547	10.13863185	9.469762946	10.89834212	7.683567165	8.084301806	7.337182439	358.2387154
ENSSSCG00000014072	5.428950457	5.796426856	5.739039804	4.329633998	4.535504033	3.951413243	338.2127894
ENSSSCG00000009022	1.866869358	2.254893125	2.234178718	1.752465972	1.446664534	1.573990112	98.9746063
ENSSSCG00000016918	1.566442228	1.834458821	1.673289164	1.37872523	1.261309987	1.175263415	135.5845248
ENSSSCG00000010370	38.73152139	36.84527004	32.07732756	23.59838141	28.36614785	29.17884351	2848.013399
ENSSSCG00000013667	1.166457609	1.674073423	1.437307001	1.017502417	1.201353025	0.978296291	119.3605243
ENSSSCG00000010654	1.609200643	1.895026276	1.279723514	1.211938121	1.318250012	1.062887885	142.4212093
ENSSSCG00000022504	1.693201813	1.511071878	1.912105671	0.982446731	1.571511042	1.276984331	150.7920195
ENSSSCG00000040366	2.404639633	2.440760228	2.179686554	1.480742154	2.175185698	1.601647426	111.8792674
ENSSSCG00000023760	1.102025201	1.330538398	1.294619474	0.924679972	0.844486926	0.990746086	102.6995858
ENSSSCG00000022447	60.84768139	64.72518605	53.01339277	41.10940471	49.27345967	42.31909537	976.910694
ENSSSCG00000017012	1.061308374	1.069496306	0.990095411	0.832597463	0.644392981	0.839964551	93.9130979
ENSSSCG00000039854	1.122425967	1.339772056	1.443631279	0.80769349	0.95738858	1.108854732	90.16206599
ENSSSCG00000031361	0.558011491	0.712828625	0.767220821	0.435976517	0.572157488	0.486952226	102.4725431
ENSSSCG00000025176	1.632428244	2.820103213	2.114077473	1.586543798	1.454075779	1.763757076	212.3388866
ENSSSCG00000024517	4.630829361	4.497846167	3.527697252	2.718324444	3.87720671	2.729681939	404.4565626
ENSSSCG00000021585	7.219159086	5.455489926	5.972924308	4.660330756	5.000481089	4.039511489	422.6947947
ENSSSCG00000039009	38.7382371	38.3868761	35.14321563	24.11209435	33.1684979	24.75824621	697.2836631
ENSSSCG00000009431	1.710880807	1.779970505	1.797521795	1.388730395	1.383613577	1.085567695	107.2666382
ENSSSCG00000038842	3.630754559	2.649954712	3.09927537	2.070765462	2.166334645	2.639310398	169.3187306
ENSSSCG00000012371	1.279637639	1.523958012	1.745199255	1.020051719	1.206012204	1.071359893	72.42839883
ENSSSCG00000035456	2.16775958	2.328783862	2.37678587	1.681222104	1.557110762	1.746477246	97.20477923
ENSSSCG00000007023	3.5436189	3.38292008	3.728467049	2.589517665	3.198285176	1.924528149	299.6758369
ENSSSCG00000017546	1.761165709	2.159685112	2.423753938	1.478254149	1.697256733	1.376879011	71.98286123
ENSSSCG00000034266	1.704232935	2.166379975	1.815746118	1.192729301	1.450460329	1.44236773	179.1255711
ENSSSCG00000009111	9.399283727	8.861516821	8.227278738	4.356713184	8.002970802	6.653500242	619.9899667
ENSSSCG00000009004	478.5621188	488.6736056	408.5516377	299.4490181	350.7377492	334.9382483	11724.05896
ENSSSCG00000012110	2.293645947	2.406383324	2.434229078	1.71574305	1.777379992	1.524437182	79.7297311
ENSSSCG00000010532	30.98303388	35.93716027	29.8382941	19.5371601	24.99339831	23.26434622	1273.669178
ENSSSCG00000016510	1.318081053	1.132740483	1.33331074	1.076853435	0.874780021	0.692970656	62.9097981
ENSSSCG00000039103	4.396636412	4.231610186	2.706871304	2.468555622	2.751397437	2.736046479	84.77030945
ENSSSCG00000006345	2.198740824	2.264506997	2.163343043	1.600297347	1.595619966	1.42186371	78.66570562
ENSSSCG00000001909	3.150243446	3.114847166	2.359132681	1.92181707	2.154391774	1.951467299	99.79585265
ENSSSCG00000029662	14.07101864	14.80985317	12.73402807	7.166759296	11.73383312	10.01764031	628.168231
ENSSSCG00000006344	1.088831647	1.025157763	1.032505171	0.609111639	0.662543253	0.912249283	75.71306662
ENSSSCG00000022446	8.712026232	8.872955985	9.020038196	5.885883166	6.565309411	5.90014192	317.849757
ENSSSCG00000006857	11.43270997	11.0737275	10.72858661	6.313194502	9.1501344	7.22163287	912.8341897

ENSSSCG00000023771	1.590671879	2.051564373	1.448438104	1.252514801	0.95367027	1.258014547	62.8224648
ENSSSCG00000004856	1.860132394	2.920254546	2.212152884	1.347657908	1.64178024	1.749235933	114.2221735
ENSSSCG00000008606	1.266518574	1.293225744	0.923917633	0.711769872	0.724939024	0.937862551	119.1858059
ENSSSCG00000006235	2.691616258	2.345791566	1.820553364	1.207354679	1.730419116	1.736128503	106.9295836
ENSSSCG00000035189	0.994300353	0.905611068	0.906682314	0.512198927	0.723082713	0.67189768	56.8612619
ENSSSCG00000037676	1.985610565	2.026904472	2.21799846	1.307540128	1.347383634	1.538364821	59.35790455
ENSSSCG00000004980	1.360116842	1.379454805	1.371194994	0.721267463	1.326581505	0.723421327	68.66509534
ENSSSCG00000008966	1.918104345	1.806808437	1.745779279	1.2504596	0.956589444	1.471468424	86.89304361
ENSSSCG00000020785	111.110316	140.9762411	113.8463227	67.16586055	87.26334889	90.66844604	3585.167952
ENSSSCG00000007574	0.779572606	0.877918827	0.680558872	0.339344543	0.678368068	0.515857762	54.36461924
ENSSSCG00000007482	2.149549452	1.96025797	2.182482597	1.7276596	1.266883731	1.112836907	68.59676873
ENSSSCG00000009667	0.735687266	0.79390059	0.649667326	0.507085021	0.551566803	0.357155907	38.95634118
Novel00098	2.175938521	1.856047396	1.691612288	0.971144548	1.39852656	1.287159185	86.86870753
ENSSSCG00000011201	0.984683497	0.948749418	0.639632934	0.388528045	0.724474187	0.525315077	38.37810062
ENSSSCG00000015036	2.359881711	2.152068158	2.683561828	1.790153412	1.344486035	1.290881781	33.66941537
ENSSSCG00000038949	0.817531947	0.905532191	0.651628735	0.48582913	0.482091392	0.488620907	55.47221413
ENSSSCG00000025924	4.694579099	5.009807709	4.285934247	2.871442698	2.864845003	2.784601059	279.331856
ENSSSCG00000009234	1.175570559	1.032573976	0.945982669	0.683634437	0.629202735	0.568811095	34.82954875
ENSSSCG00000005278	10.50012333	8.756227319	8.815770158	4.645208351	7.248923952	4.555544858	431.3628852
Novel00315	1.237483513	1.540849472	1.235180059	0.632539009	0.772520068	0.888242723	67.1397763
ENSSSCG00000034943	1.266511851	1.333393329	1.514244521	0.514965136	0.861751025	0.942637334	19.03704617
ENSSSCG00000041935	0.618222533	0.909737903	0.995506841	0.689823495	0.27997582	0.412052339	43.46389333
ENSSSCG00000026520	0.656132405	0.632188149	0.690717504	0.400664871	0.261486851	0.418933886	26.26323277
ENSSSCG00000004632	0.898247683	0.822194513	0.693778803	0.329107012	0.44747058	0.548801258	31.41390689
ENSSSCG00000016403	0.924414595	0.951621167	0.841755827	0.405541799	0.610776212	0.43865502	15.15135575
ENSSSCG00000000040	0.896202552	0.699887375	1.133425601	0.584707135	0.307033504	0.414363484	20.99379181
Novel00024	1.218648922	0.665366818	1.045829818	0.446498124	0.566609388	0.362216627	11.99947485
ENSSSCG00000032334	3.21475717	2.835349799	3.030508035	1.427008005	0.689860421	1.029017191	6.429522577
ENSSSCG00000036779	0.606100522	8.175749131	6.553866858	1.554474951	0	1.68140064	2.697600871
ENSSSCG00000018359	2.72745235	2.943269687	3.932320115	0	0.760875464	0	0.354675162

readcount_NC	log2FoldChange	pval		padj	significant	chromosome	start	end	length
0	5.1091	0.0018449	up	NA	TRUE	6	7650444	7661320	390
0.343979049	3.5465	0.046408	up	NA	TRUE	6	166552885	166552964	80
2.943253889	1.7749	0.045124	up	NA	TRUE	1	56378291	56395493	973
5.143595551	1.5921	0.036625	up	NA	TRUE	11	15550742	15603974	1327
6.892220455	1.4764	0.016444	up	NA	TRUE	3	92673469	92695954	1114
6.392844025	1.4462	0.031527	up	NA	TRUE	5	64058501	64059604	1104
40.75350305	1.3995	8.70E-07	up	0.0011796	TRUE	1	91092287	91097170	1901
55.51657097	1.3654	0.017269	up	0.6186	TRUE	15	129172242	129175941	1192
8.459223508	1.3072	0.034108	up	NA	TRUE	2	78730389	78752870	937
18.19174941	1.2678	0.0040148	up	NA	TRUE	12	804718	806057	1197
17.15981226	1.2584	0.0033222	up	NA	TRUE	14	15718266	15763710	2386
8.709079043	1.2334	0.035023	up	NA	TRUE	9	91600207	91600280	74
19.93756369	1.2047	0.010744	up	NA	TRUE	9	10413187	10435100	2080
10.6273556	1.2015	0.020995	up	NA	TRUE	2	44245377	44249053	566
11.15262186	1.1706	0.049524	up	NA	TRUE	4	67847352	67847440	89
21.53296176	1.1505	0.002908	up	NA	TRUE	6	88691869	88696057	2082
40.93392907	1.0564	0.0049173	up	0.37394	TRUE	17	56674872	56683261	3006
16.091851	1.0451	0.026287	up	NA	TRUE	X	67706306	67713580	2068
221.2937749	1.0253	0.022843	up	0.67437	TRUE	18	50002925	50010690	2362
29.25623286	1.0236	0.0028077	up	0.29028	TRUE	12	808153	814703	6292
70.01636108	0.99587	2.47E-05	up	0.014864	FALSE	14	48040881	48051660	4658
338.9838414	0.97689	0.0023851	up	0.27041	FALSE	11	18953817	18981005	2481
733.6056258	0.96755	0.00024683	up	0.065278	FALSE	8	70054888	70159145	1120
31.76617345	0.96546	0.026391	up	0.6913	FALSE	6	59554323	59554859	537
31.71970961	0.94204	0.0059971	up	0.40155	FALSE	6	59548440	59553909	4637
19.59639527	0.93712	0.034492	up	NA	FALSE	2	32623268	32679164	1452
1391.755106	0.9353	2.19E-12	up	2.37E-08	FALSE	8	69932643	69935861	1702
22.24149387	0.92413	0.02079	up	NA	FALSE	9	98437087	98513156	4739
9958.25097	0.87374	6.47E-05	up	0.026987	FALSE	8	70008162	70010358	1550
3343.353447	0.86734	0.002363	up	0.27041	FALSE	6	59541210	59544896	1200
310.3817715	0.85401	0.023149	up	0.67838	FALSE	14	7950389	7961566	2292
24.44434072	0.83104	0.042219	up	NA	FALSE	7	98449349	98455554	2605
60.97377191	0.8199	0.0023681	up	0.27041	FALSE	13	125337417	125338850	602
357.0980581	0.81261	1.02E-05	up	0.007353	FALSE	9	91506340	91511263	956
40.20515759	0.80261	0.0093516	up	0.49763	FALSE	6	115505710	115592726	4749
1366.636178	0.79313	0.0016769	up	0.23312	FALSE	3	43718368	43731119	1689

52.42794046	0.79268	0.0054778	up	0.40034	FALSE	1		223385097	223549757	4372
811.3208494	0.78592	0.0032886	up	0.31232	FALSE	2		137803988	137812914	1514
36.29820023	0.77494	0.02248	up	0.67064	FALSE	1		91443754	91448733	3624
82.75591993	0.77199	0.00058908	up	0.10646	FALSE	6		78824284	78879462	1070
562.2573878	0.76407	0.010467	up	0.5159	FALSE	1		157872212	157886753	1806
139.7226551	0.75182	7.27E-05	up	0.02826	FALSE	5		81540231	81550969	5001
48.07219051	0.73742	0.018064	up	0.62778	FALSE	3		64878846	64880270	903
41.28355858	0.7336	0.023775	up	0.67983	FALSE	14		113420556	113434349	1395
1595.734873	0.73182	0.0044297	up	0.35844	FALSE	9		127850013	127858884	3707
171.4432259	0.72887	4.38E-05	up	0.022624	FALSE	4		29933135	30233960	3016
70.1797222	0.726	0.0073897	up	0.46789	FALSE	15		55490468	55509459	5213
114.9763153	0.72515	0.00018738	up	0.053469	FALSE	6		59532093	59539954	2859
57.34701456	0.72132	0.030891	up	0.71724	FALSE	3		122716974	122731929	2729
36.80737631	0.7127	0.042619	up	0.78536	FALSE	1		71380768	71400192	1502
294.3289472	0.71018	1.47E-05	up	0.0094026	FALSE	1		73120508	73124342	860
135.8696187	0.67859	0.00051943	up	0.098894	FALSE	15		132931724	132997750	3368
245.930788	0.6711	7.97E-05	up	0.029812	FALSE	17		41186766	41221637	4204
73.44735584	0.66959	0.0046447	up	0.37014	FALSE	8		936688	938485	1524
55.63942415	0.66802	0.024377	up	0.68141	FALSE	13		108517310	108550382	6418
37.13905052	0.66765	0.04911	up	0.82759	FALSE	15		121329288	121349276	3277
482.8249428	0.65739	7.55E-06	up	0.006294	FALSE	1		241569905	241611734	3375
419.4599927	0.65146	2.81E-07	up	0.00050694	FALSE	9		102868836	102875577	4486
48.86624232	0.63989	0.02198	up	0.67064	FALSE	1		203699251	203699752	417
52.32248683	0.62307	0.032706	up	0.73119	FALSE	11		4746403	4748894	1087
119.4034994	0.62073	0.0076427	up	0.47354	FALSE	6		93833759	93834920	820
192.1519049	0.6179	0.0024631	up	0.27041	FALSE	11		50072554	50102884	3025
59.20109309	0.61716	0.024242	up	0.68141	FALSE	12		58126044	58168407	5365
182.5772232	0.61304	0.00036331	up	0.080395	FALSE	6		69507825	69535372	2152
76.16447348	0.61276	0.022636	up	0.67243	FALSE	14		46400026	46408366	2556
97.80954324	0.61061	0.003475	up	0.31463	FALSE	16		49188936	49193709	3778
559.4910184	0.60452	3.68E-05	up	0.019973	FALSE	12		796884	803554	2231
252.9465018	0.57727	8.81E-05	up	0.030424	FALSE	4		117496908	117511226	2445
71.49970955	0.57614	0.016094	up	0.60027	FALSE	2		27044737	27055453	1702
85.47022693	0.5697	0.016324	up	0.6041	FALSE	13		207118812	207132538	1840
62.91128393	0.5639	0.048372	up	0.82752	FALSE	12		20159773	20170776	4851
2992.745585	0.56093	2.29E-06	up	0.002262	FALSE	10		27668264	27674618	1590
115.0862528	0.56015	0.0068231	up	0.44301	FALSE	13		148090629	148192231	3788

61.57456632	0.55843	0.041341	up	0.77906	FALSE	X	106236748	106246378	2995
272.6437333	0.55762	0.00084007	up	0.14162	FALSE	11	52153704	52160497	2078
62.19395609	0.55544	0.048373	up	0.82752	FALSE	11	1029308	1066085	1655
105.8745936	0.55441	0.012228	up	0.54356	FALSE	3	39580200	39584386	1589
61.63300035	0.55301	0.048876	up	0.82759	FALSE	18	18006396	18011337	4404
144.6163659	0.55008	0.0037542	up	0.3299	FALSE	AEMK02000682.1	813245	820123	3692
431.8817678	0.5385	0.00028922	up	0.06827	FALSE	8	70417923	70428102	2467
210.9544403	0.53665	0.0017267	up	0.23396	FALSE	15	63487698	63505244	2395
79.75456664	0.53324	0.018759	up	0.63248	FALSE	7	13947304	14586092	5639
74.21835765	0.53162	0.024509	up	0.68141	FALSE	7	19587685	19838093	4262
94.39912249	0.52938	0.011043	up	0.52941	FALSE	4	98800930	98814553	2676
178.4488896	0.52806	0.0057565	up	0.40034	FALSE	4	618988	621343	1287
103.521389	0.52594	0.028286	up	0.70929	FALSE	5	21147767	21160738	859
827.2730785	0.52563	1.00E-05	up	0.007353	FALSE	1	269911789	269924462	1124
66.39845785	0.52388	0.036719	up	0.75195	FALSE	4	12817346	12828282	4479
173.9812268	0.52184	0.0014178	up	0.20228	FALSE	X	86785325	86814629	4402
99.12635612	0.52119	0.012901	up	0.54947	FALSE	5	22535740	22549354	1327
71.75271035	0.52076	0.040846	up	0.77309	FALSE	5	19245851	19248172	1623
168.3466084	0.51487	0.016571	up	0.611	FALSE	4	90370235	90401376	2272
1975.327882	0.51309	1.27E-07	up	0.0003453	FALSE	12	40798109	40800028	759
186.8834483	0.5126	0.00093128	up	0.14426	FALSE	8	72791704	72800551	2941
158.2801534	0.50322	0.0047109	up	0.37014	FALSE	4	87286953	87344251	3601
174.7408985	0.50091	0.0033988	up	0.31232	FALSE	4	61114497	61142811	6507
1066.03337	0.49535	8.58E-05	up	0.030424	FALSE	13	190361449	190412949	6300
83.60626442	0.49266	0.032618	up	0.73119	FALSE	7	23050195	23064498	2577
369.563847	0.4855	0.0029257	up	0.29648	FALSE	5	17385390	17408514	2682
86.03970216	0.48377	0.038845	up	0.75756	FALSE	14	132240887	132247816	494
303.1069518	0.48245	0.019734	up	0.64263	FALSE	AEMK02000452.1	3029790	3038173	1455
594.0985012	0.47927	0.0031068	up	0.30601	FALSE	13	206454718	206465807	4445
123.3543602	0.47797	0.011716	up	0.54169	FALSE	9	77914958	78216736	1076
93.02167583	0.47559	0.038162	up	0.75579	FALSE	9	96065922	96387950	4084
220.8045295	0.47003	0.011482	up	0.53627	FALSE	12	60880592	60919279	1269
97.66993061	0.46784	0.029071	up	0.70929	FALSE	9	76699561	76772401	1216
101.625857	0.46654	0.024139	up	0.68141	FALSE	9	75246002	75260134	4092
1332.586225	0.46481	0.00016086	up	0.049833	FALSE	14	140362127	140371881	1772
1860.454853	0.46398	1.77E-07	up	0.00038275	FALSE	8	101278878	101343916	6437
197.5740272	0.46116	0.0088156	up	0.49041	FALSE	14	29886042	29894847	2581

90.96214263	0.45646	0.038148	up	0.75579	FALSE	1	36417053	36695954	4114
100.3864082	0.45364	0.032023	up	0.72794	FALSE	9	12585955	12662957	4297
312.6589707	0.4516	0.00067142	up	0.11935	FALSE	16	34183546	34194578	2474
149.8925007	0.45151	0.013746	up	0.56032	FALSE	X	110159912	110170350	1219
376.0474422	0.45049	0.00025885	up	0.066255	FALSE	X	110384592	110387902	1218
123.868631	0.44982	0.040925	up	0.77309	FALSE	14	50234156	50251642	1823
1454.245222	0.44876	0.00030644	up	0.070696	FALSE	9	127859051	128162636	3061
161.6020719	0.44685	0.010348	up	0.5159	FALSE	5	15300451	15307697	2907
538.466817	0.44607	0.0031992	up	0.30698	FALSE	13	190276424	190286205	4473
2648.715745	0.44205	5.84E-05	up	0.026375	FALSE	9	21722035	21761473	4491
114.8113686	0.44195	0.034687	up	0.7376	FALSE	4	117635861	117805254	3192
258.6661977	0.43499	0.0018262	up	0.24149	FALSE	2	76648084	76655814	1883
1249.733124	0.43297	0.0018745	up	0.24196	FALSE	14	47221560	47238749	4516
433.2381398	0.42033	0.0022244	up	0.27041	FALSE	9	77910881	77937060	1148
168.8553947	0.41889	0.035466	up	0.73811	FALSE	4	12126855	12409055	2766
455.5119016	0.41679	0.0056518	up	0.40034	FALSE	4	75525555	75561444	1612
222.2145203	0.41407	0.02593	up	0.6913	FALSE	6	54646574	54675115	6566
1160.737104	0.41232	7.30E-05	up	0.02826	FALSE	3	127500759	127503257	1332
1559.343109	0.41229	0.00028963	up	0.06827	FALSE	2	120117652	120193715	4084
162.6185589	0.41026	0.014806	up	0.58629	FALSE	6	85596981	85597846	396
135.2034305	0.41008	0.037348	up	0.75412	FALSE	17	52743570	52906821	7037
346.5512962	0.40686	0.011015	up	0.52941	FALSE	5	9683658	9696025	2137
128.0105496	0.40576	0.031782	up	0.72475	FALSE	14	127717665	127721420	2215
184.0747967	0.40423	0.018785	up	0.63248	FALSE	2	60450425	60459121	3446
233.4448701	0.40361	0.011963	up	0.54356	FALSE	2	25420665	25423166	2502
262.5589851	0.40327	0.03144	up	0.72475	FALSE	7	30320454	30329409	1866
359.6425124	0.40277	0.0017477	up	0.23396	FALSE	5	73411038	73497180	5286
155.9099098	0.40227	0.036245	up	0.74898	FALSE	4	74612527	74745989	2333
340.7598283	0.40214	0.0046811	up	0.37014	FALSE	2	59166126	59193948	3734
216.2674372	0.40027	0.03035	up	0.71323	FALSE	12	60922473	60924255	1621
139.4658105	0.39363	0.036364	up	0.7496	FALSE	15	139978349	139981875	2965
206.880697	0.39355	0.025357	up	0.6913	FALSE	3	41134246	41141267	3113
323.679024	0.39182	0.013381	up	0.55805	FALSE	17	46933503	47044049	1472
216.4957148	0.39157	0.023821	up	0.67983	FALSE	3	1052191	1054688	770
180.5686376	0.39111	0.015596	up	0.59545	FALSE	18	42549846	42651807	5927
366.8949156	0.39061	0.0019741	up	0.24358	FALSE	1	57128305	57137085	3253
176.3875205	0.38918	0.04889	up	0.82759	FALSE	8	118151957	118164643	1635

368.8484394	0.38915	0.011183	up	0.52941	FALSE	17	48175056	48188264	3271
2271.915374	0.38762	0.00014284	up	0.045553	FALSE	6	58860184	58886884	5037
463.9027779	0.38696	0.0017331	up	0.23396	FALSE	10	936789	940098	1211
196.916079	0.38554	0.040219	up	0.76899	FALSE	12	991757	994745	1620
900.8250009	0.38249	0.0034821	up	0.31463	FALSE	6	53470787	53474652	655
921.7699445	0.38105	0.00018334	up	0.053469	FALSE	9	86511369	86555943	5398
212.1228156	0.37553	0.018534	up	0.63248	FALSE	7	29723774	29753934	6042
1994.669007	0.3711	0.0085788	up	0.49041	FALSE	15	118822047	118850976	1421
496.5988889	0.37016	0.0055967	up	0.40034	FALSE	1	166465953	166608367	3917
18947.33884	0.36787	0.00013146	up	0.043195	FALSE	X	42102172	42106297	812
205.6854572	0.36686	0.034541	up	0.7376	FALSE	7	23914603	23928196	3583
722.9738647	0.36619	0.00027246	up	0.067143	FALSE	12	31695849	31723726	3131
167.1856351	0.3654	0.036814	up	0.75195	FALSE	14	7360035	7371718	3689
462.9057242	0.36388	0.0125	up	0.54356	FALSE	6	71825366	71837572	2252
422.8234457	0.36274	0.035121	up	0.7376	FALSE	AEMK02000682.1	965800	977176	6418
164.4218325	0.36128	0.045804	up	0.80993	FALSE	13	72573332	72579192	2273
709.423553	0.36094	0.0042177	up	0.35264	FALSE	7	23265562	23267042	1385
165.7804043	0.35897	0.047694	up	0.82218	FALSE	13	40009737	40055490	2225
261.7096737	0.35876	0.02763	up	0.70492	FALSE	14	47392665	47396733	1002
632.2412015	0.35756	0.0094388	up	0.49924	FALSE	13	206676730	206703327	5830
4097.946782	0.35536	0.00045626	up	0.093343	FALSE	9	115857644	115868892	1441
576.7964868	0.35371	0.014087	up	0.56861	FALSE	6	63670753	63675839	765
3187.242956	0.34957	3.52E-05	up	0.019973	FALSE	14	4104772	4135814	3083
522.1127473	0.34363	0.01174	up	0.54169	FALSE	9	64190982	64207006	3885
514.4801165	0.3417	0.014245	up	0.56997	FALSE	7	47929035	47994486	5461
264.5479162	0.34111	0.023888	up	0.67983	FALSE	2	26095038	26115464	926
214.3086527	0.33813	0.042817	up	0.78536	FALSE	3	39942366	39952825	2241
322.0961519	0.33725	0.011524	up	0.53627	FALSE	X	107992	155864	2035
220.6067331	0.33506	0.044034	up	0.79168	FALSE	10	64776315	64859051	6132
786.3003699	0.33422	0.0033865	up	0.31232	FALSE	2	6922387	6950179	2948
362.3719116	0.33271	0.041892	up	0.78237	FALSE	6	964566	976832	3218
197.7624954	0.32769	0.044021	up	0.79168	FALSE	2	142102456	142115344	2811
755.6348154	0.32759	0.024414	up	0.68141	FALSE	12	36878675	36887513	2746
315.2433479	0.32679	0.031198	up	0.72128	FALSE	13	133968836	133970495	886
306.9980142	0.32672	0.021022	up	0.65501	FALSE	6	105613273	105645113	2400
272.8122675	0.3241	0.02935	up	0.70929	FALSE	4	54801368	54838143	3085
620.0773824	0.32123	0.0029135	up	0.29648	FALSE	15	81931383	81935529	2844

4688.494099	0.3188	0.0041929	up	0.35264	FALSE	17	35315964	35317744	1554
311.9526934	0.31849	0.020403	up	0.64688	FALSE	6	52476117	52477532	1416
665.0675676	0.31832	0.0059665	up	0.40155	FALSE	8	81209327	81276853	4918
715.0176403	0.31701	0.025687	up	0.6913	FALSE	2	6797358	6803782	3808
534.5946263	0.31696	0.022887	up	0.67437	FALSE	13	206111408	206127732	6143
752.1391745	0.31655	0.01896	up	0.63257	FALSE	6	68669153	68682624	1617
593.0699278	0.31595	0.0063742	up	0.41888	FALSE	X	26470417	26557262	3735
9375.336868	0.31205	0.008778	up	0.49041	FALSE	12	21961645	21980211	4153
351.8981958	0.31064	0.029418	up	0.70929	FALSE	6	56011491	56018170	1245
1358.602454	0.30988	0.0048968	up	0.37394	FALSE	6	68954771	69258631	4575
238.3208759	0.30805	0.038527	up	0.75588	FALSE	14	113809412	113868270	2586
1825.234195	0.30801	0.0024938	up	0.27041	FALSE	8	39702613	39708167	1858
1054.734929	0.3063	0.01123	up	0.52941	FALSE	7	23004251	23015415	1576
5454.771682	0.30594	0.00035811	up	0.080395	FALSE	1	122215878	122542224	3606
288.2019905	0.30491	0.024959	up	0.68871	FALSE	6	85533589	85538276	2595
522.1948714	0.29981	0.0083587	up	0.48467	FALSE	7	23253160	23265106	1627
1207.961179	0.29979	0.0014524	up	0.20452	FALSE	X	19907900	19910895	1157
399.6322497	0.29927	0.026539	up	0.6913	FALSE	10	57829518	57895610	1920
463.3942971	0.29877	0.023719	up	0.67983	FALSE	12	15435964	15447101	2548
1079.679231	0.29834	0.0073496	up	0.46789	FALSE	16	37897300	39154586	6799
633.8848701	0.29775	0.034281	up	0.7376	FALSE	3	59189177	59264049	1653
268.1049094	0.29593	0.034474	up	0.7376	FALSE	17	9536738	9541384	2495
717.5477618	0.29573	0.0093623	up	0.49763	FALSE	10	65705045	65705590	546
469.785669	0.29508	0.033498	up	0.7376	FALSE	17	32629282	32631430	1050
590.1329152	0.29413	0.020096	up	0.64263	FALSE	6	146166681	146720127	4965
338.090286	0.29412	0.024459	up	0.68141	FALSE	11	20555961	20619629	5202
1154.647789	0.29383	0.025959	up	0.6913	FALSE	13	207106206	207115920	2009
575.6412775	0.29322	0.039477	up	0.76301	FALSE	12	47791911	47838946	7697
753.9548172	0.29165	0.031667	up	0.72475	FALSE	2	76146988	76155759	8516
1978.031446	0.29144	0.0058834	up	0.40155	FALSE	2	49229223	49231866	1838
288.8175657	0.2912	0.03427	up	0.7376	FALSE	14	111388898	111397622	2489
471.2905311	0.29083	0.012193	up	0.54356	FALSE	4	95713563	95716154	769
3794.902636	0.28232	0.012633	up	0.54356	FALSE	6	4333731	4399237	1186
532.4769386	0.28148	0.018666	up	0.63248	FALSE	15	104781615	104844921	3779
327.0532779	0.28106	0.0397	up	0.76528	FALSE	1	272970548	272975293	1228
386.7598202	0.28051	0.034335	up	0.7376	FALSE	1	83847812	83850881	2475
405.8615439	0.27782	0.037797	up	0.75579	FALSE	2	89208742	89255449	1313

3436.443067	0.27717	0.0062017	up	0.41003	FALSE	13	124955029	125054463	6106
988.7318952	0.27705	0.020045	up	0.64263	FALSE	2	75492207	75501543	1663
437.5234271	0.27616	0.043264	up	0.78573	FALSE	1	254012196	254027485	4494
11646.20631	0.27385	0.0013942	up	0.20157	FALSE	15	132355655	132361435	1036
3018.680378	0.27121	0.00050116	up	0.098801	FALSE	8	12624109	12635334	3577
405.1348846	0.27041	0.049008	up	0.82759	FALSE	2	65062766	65072681	1831
519.2446869	0.27021	0.019083	up	0.63279	FALSE	15	862013	881461	1519
2070.308491	0.27018	0.0055971	up	0.40034	FALSE	5	82290382	82325563	1800
966.8842916	0.26979	0.03855	up	0.75588	FALSE	6	55316471	55322250	1617
612.9775076	0.2695	0.01611	up	0.60027	FALSE	7	98574198	98616474	2086
560.4500986	0.26871	0.02384	up	0.67983	FALSE	13	131135724	131138236	1471
2718.176672	0.26813	0.00087168	up	0.14174	FALSE	7	92490011	92495574	3084
1119.194807	0.26729	0.0038882	up	0.33728	FALSE	6	160108161	160136168	2870
426.9065696	0.26668	0.038268	up	0.75581	FALSE	2	129880658	130026419	1409
602.3374282	0.26638	0.018841	up	0.63248	FALSE	1	161182868	161188164	2694
401.8409189	0.26601	0.039192	up	0.75988	FALSE	8	47009463	47098718	1823
375.8114771	0.26536	0.040298	up	0.76899	FALSE	7	94157731	94219385	3339
504.384961	0.26361	0.033622	up	0.7376	FALSE	6	17632494	17645806	2778
774.4452302	-0.26306	0.026693	down	0.6913	FALSE	6	19659987	19730359	3757
4879.340232	-0.26338	0.0080884	down	0.48467	FALSE	X	46282415	46441623	13394
2809.101714	-0.26411	0.0079348	down	0.48467	FALSE	3	110823666	110843068	2470
1344.290495	-0.2649	0.042978	down	0.78536	FALSE	1	77197409	77400137	9599
986.8386413	-0.26544	0.010262	down	0.5159	FALSE	1	109208672	109680199	8518
1511.157378	-0.26574	0.0082453	down	0.48467	FALSE	4	50370088	50509113	4961
2938.11761	-0.26716	0.0013449	down	0.19706	FALSE	7	53498963	53519688	6123
847.398386	-0.26732	0.022155	down	0.67064	FALSE	1	169373888	169596636	7904
14627.00506	-0.26822	0.00070251	down	0.12091	FALSE	13	70757265	70830388	4060
67367.97791	-0.26901	0.00057787	down	0.1062	FALSE	16	71357703	71381160	2167
694.2929003	-0.2691	0.046538	down	0.813	FALSE	9	45743431	45825195	13220
1585.514763	-0.27065	0.0055014	down	0.40034	FALSE	17	50925789	50958705	7279
735.1127075	-0.27091	0.034474	down	0.7376	FALSE	16	52836294	53160162	4452
1030.16926	-0.2711	0.013437	down	0.55821	FALSE	7	49258910	49450463	6797
3377.970779	-0.27134	0.012464	down	0.54356	FALSE	16	3563660	3921556	8074
920.6519519	-0.27134	0.023765	down	0.67983	FALSE	13	125782471	126489650	3678
5043.897934	-0.27139	0.0025747	down	0.27641	FALSE	4	94026385	94080422	4666
1494.983289	-0.27201	0.00487	down	0.37394	FALSE	17	34807534	34816451	3406
610.9908897	-0.2725	0.020571	down	0.64724	FALSE	4	8078156	8110829	3089

1187.89594	-0.27272	0.0042574	down	0.35264	FALSE	7	100181012	100197836	3619
2991.107842	-0.27337	0.014778	down	0.58629	FALSE	9	126007493	126172579	5086
2602.00851	-0.27414	0.016679	down	0.611	FALSE	1	20511240	21048787	10943
669.1607934	-0.27527	0.014124	down	0.56861	FALSE	14	2804061	3035241	3788
2148.307156	-0.27565	0.0089781	down	0.49041	FALSE	4	101922251	102032277	3911
1126.326635	-0.27602	0.0073245	down	0.46789	FALSE	8	96118610	96692407	4575
3538.105774	-0.27613	0.015168	down	0.58934	FALSE	18	19759738	19787618	9042
2430.191185	-0.2768	0.03314	down	0.73634	FALSE	16	43663002	44080499	5876
3283.533482	-0.27849	0.0018637	down	0.24196	FALSE	12	20407233	20472993	4945
657.4229115	-0.27918	0.012371	down	0.54356	FALSE	11	78950878	79027930	4912
12600.76893	-0.27922	0.0030727	down	0.30601	FALSE	12	53647576	53781557	5004
801.3889907	-0.28015	0.018903	down	0.63257	FALSE	1	139494120	139624605	7269
467.3993585	-0.28087	0.040233	down	0.76899	FALSE	1	188335384	188511096	4515
395.7446997	-0.28152	0.045347	down	0.80872	FALSE	6	66928444	67016836	2822
526.6986983	-0.28532	0.031816	down	0.72475	FALSE	14	111307610	111351712	1783
702.3462016	-0.28553	0.017931	down	0.62518	FALSE	14	32623308	32664598	4979
10274.27004	-0.28652	0.0012662	down	0.18807	FALSE	1	99081938	99110876	3760
751.9113683	-0.28659	0.0083083	down	0.48467	FALSE	17	48231495	48263944	3144
859.3402963	-0.28674	0.010027	down	0.5159	FALSE	12	38941089	38978448	5862
2873.413935	-0.29131	0.012046	down	0.54356	FALSE	2	116812582	117034912	8789
645.541489	-0.29204	0.01041	down	0.5159	FALSE	1	163089444	163103446	2323
1025.953704	-0.29345	0.010176	down	0.5159	FALSE	6	89006672	89026710	2585
537.7457298	-0.29418	0.02692	down	0.6913	FALSE	15	47730506	47861057	2682
1301.510766	-0.29501	0.01201	down	0.54356	FALSE	7	37655847	37784359	6377
5554.227702	-0.29532	0.013643	down	0.55997	FALSE	5	11264590	11422660	1334
731.3102902	-0.296	0.008261	down	0.48467	FALSE	4	84597425	84700186	1524
834.4941274	-0.29624	0.0056516	down	0.40034	FALSE	12	44616916	44629934	7258
1148.418076	-0.29904	0.0049661	down	0.37394	FALSE	18	42924727	42987924	5243
971.4577665	-0.30007	0.024962	down	0.68871	FALSE	3	26653792	26754882	10903
501.6794822	-0.30073	0.03413	down	0.7376	FALSE	14	45320588	45925027	8481
2162.081066	-0.30272	0.015657	down	0.59566	FALSE	14	60667108	60677731	3691
2457.925408	-0.30275	0.00053775	down	0.10053	FALSE	7	38603474	38613448	3204
10432.26831	-0.30292	0.00039463	down	0.083901	FALSE	7	49622536	49792543	6308
3150.463902	-0.30306	0.012608	down	0.54356	FALSE	9	95280593	95486104	4640
2291.283635	-0.3053	0.0041312	down	0.35264	FALSE	11	67764009	68130255	7913
981.8363548	-0.30563	0.010233	down	0.5159	FALSE	14	34409070	34513025	4964
961.3136155	-0.30588	0.002407	down	0.27041	FALSE	16	52003735	52166161	4352

390.5843739	-0.30772	0.020209	down	0.64263	FALSE	18		42432050	42512266	6605
2723.098525	-0.30794	0.0012059	down	0.18416	FALSE	13		147179591	147248861	7732
2535.534352	-0.30876	0.0096234	down	0.50654	FALSE	10		50540556	50680154	7256
423.959225	-0.30905	0.01987	down	0.64263	FALSE	6		77406372	77456229	5435
470.2923924	-0.30923	0.026748	down	0.6913	FALSE	11		8805913	8858418	10304
1143.180299	-0.30964	0.0040036	down	0.34453	FALSE	1		145508636	145697009	3607
669.3088995	-0.30976	0.018227	down	0.63143	FALSE	3		69253748	69417397	12854
1807.795681	-0.31025	0.0052669	down	0.39116	FALSE	2		60455605	60567505	7608
698.0176189	-0.31185	0.012577	down	0.54356	FALSE	1		193911965	194264354	21367
1222.494098	-0.31243	0.0031563	down	0.30601	FALSE	1		14028858	14201706	6674
2593.682192	-0.31376	0.011824	down	0.54324	FALSE	13		138754810	138847249	11177
1498.486972	-0.31455	0.0098466	down	0.51522	FALSE	8		38397375	38697206	9893
1358.777259	-0.31819	0.0076408	down	0.47354	FALSE	13		175505366	176479482	6186
1933.982905	-0.32077	0.02021	down	0.64263	FALSE	3		11214073	11244897	2221
939.7687938	-0.32455	0.002811	down	0.29028	FALSE	1		2203605	2522604	5154
3127.955747	-0.32467	0.00090198	down	0.14174	FALSE	14		111461560	111478031	5354
699.8914946	-0.326	0.013113	down	0.54947	FALSE	1		127958518	128028718	6034
486.5712946	-0.3263	0.034843	down	0.7376	FALSE	8		116415267	116545299	5017
273.0946673	-0.3291	0.049153	down	0.82759	FALSE	11		8343265	8765082	10287
374.2033001	-0.32938	0.045992	down	0.80993	FALSE	13		46896518	47567518	6203
1066.668483	-0.32992	0.0036279	down	0.32243	FALSE	1		272095358	272170750	8422
1773.606871	-0.333	8.98E-05	down	0.030424	FALSE	6		34519180	34585779	4561
277.3488041	-0.33586	0.048386	down	0.82752	FALSE	18		16052663	16973768	4567
1044.096848	-0.33681	0.00051987	down	0.098894	FALSE	17		31072738	31217342	4537
251.9312105	-0.33681	0.037792	down	0.75579	FALSE	9		72385263	72444616	4105
724.0313019	-0.33733	0.006167	down	0.41003	FALSE	7		120703313	120890794	4155
200.7238181	-0.33895	0.049584	down	0.82931	FALSE	6		62797139	62818472	4020
546.5034373	-0.33914	0.010422	down	0.5159	FALSE	13		115317263	115488176	2171
456.6952756	-0.33996	0.037081	down	0.75195	FALSE	6		65053073	65141497	6607
314.8873435	-0.34387	0.026436	down	0.6913	FALSE	13		17651165	18031518	3546
287.8522766	-0.34445	0.026609	down	0.6913	FALSE	6		93839174	93884405	3860
233.3162529	-0.3466	0.043189	down	0.78573	FALSE	1		253428508	253550375	2103
249.4219392	-0.34836	0.029664	down	0.70956	FALSE	7		9105802	9675230	3931
200.2790612	-0.34879	0.046742	down	0.81351	FALSE	14		77375758	77597674	5609
441.5112317	-0.35214	0.0086254	down	0.49041	FALSE	8		74664598	74934837	10814
387.4414585	-0.35304	0.013125	down	0.54947	FALSE	14		108304025	108436999	7412
3701.242706	-0.35374	0.00040265	down	0.083961	FALSE	11		49164141	49432858	14516

405.0686588	-0.3552	0.022037	down	0.67064	FALSE	14	41968466	42025726	4856
243.30267	-0.35523	0.029325	down	0.70929	FALSE	X	82761589	82774952	2053
204.7691079	-0.35524	0.045503	down	0.80993	FALSE	14	48421880	48523595	5314
296.5754672	-0.36026	0.028804	down	0.70929	FALSE	12	14582481	14723336	4263
3520.699922	-0.36047	5.14E-05	down	0.025357	FALSE	2	70182925	70197384	1859
237.4597863	-0.36362	0.026497	down	0.6913	FALSE	12	23688699	23723377	6117
630.1245376	-0.36509	0.0054457	down	0.40034	FALSE	15	59672538	59709380	2005
486.1127315	-0.36869	0.012486	down	0.54356	FALSE	13	61068126	61398908	7516
1202.064109	-0.3709	0.0057598	down	0.40034	FALSE	8	104498710	104560509	6276
464.2044423	-0.37112	0.0058113	down	0.40135	FALSE	12	12882064	13263554	2927
239.0827766	-0.37565	0.035423	down	0.73811	FALSE	13	22125121	22250466	5331
452.2531421	-0.37595	0.015779	down	0.59614	FALSE	7	40980523	41227021	2488
944.8579916	-0.37777	0.0052273	down	0.3909	FALSE	13	121637866	121746129	5902
162.0073248	-0.37822	0.046279	down	0.81197	FALSE	3	71730002	71840862	2568
224.9589739	-0.37914	0.036219	down	0.74898	FALSE	13	122467588	122488293	4657
5917.695012	-0.38033	0.00037961	down	0.082323	FALSE	10	46132914	46303365	5630
1613.810929	-0.38035	0.0024555	down	0.27041	FALSE	1	229578568	229581017	2274
1167.7561	-0.38705	0.0019129	down	0.24358	FALSE	9	126850148	127342685	18124
393.9921208	-0.38831	0.024279	down	0.68141	FALSE	13	162355469	162478720	9522
212.0637415	-0.38873	0.026968	down	0.6913	FALSE	18	24372544	24867284	4676
643.8149945	-0.39099	0.004282	down	0.35264	FALSE	15	139610626	139694826	6975
192.9372945	-0.39099	0.042055	down	0.78237	FALSE	1	97610691	98015089	4200
495.1963552	-0.39139	0.0012415	down	0.18696	FALSE	6	120036838	120586849	9662
283.832376	-0.3951	0.033893	down	0.7376	FALSE	14	6834779	6848531	1449
287.8809771	-0.40096	0.018446	down	0.63248	FALSE	14	62565138	62925849	3603
295.6913276	-0.40404	0.018803	down	0.63248	FALSE	8	108726113	108803559	1917
331.1072592	-0.40567	0.030421	down	0.71323	FALSE	1	13972615	14024472	6362
1496.122493	-0.40737	0.0001866	down	0.053469	FALSE	12	38581449	38875134	9272
180.6084763	-0.41297	0.042697	down	0.78536	FALSE	4	130689025	130701683	4174
888.794524	-0.41453	0.00048351	down	0.097086	FALSE	8	23459475	23872006	4103
141.5053255	-0.41492	0.043718	down	0.79006	FALSE	6	119104767	119132588	4780
203.5885905	-0.416	0.034529	down	0.7376	FALSE	7	86777920	87103309	2849
240.3544057	-0.41687	0.0098834	down	0.51522	FALSE	13	140666132	140671017	4737
263.5513631	-0.41821	0.0089327	down	0.49041	FALSE	7	30731461	30802735	4502
188.496886	-0.42139	0.024787	down	0.68739	FALSE	12	40481784	40494148	3117
1087.877592	-0.42151	0.00022824	down	0.063457	FALSE	8	109306793	109821078	5818
379.773447	-0.4224	0.0019539	down	0.24358	FALSE	8	47702578	47838521	6645

480.903102	-0.42503	0.00088673	down	0.14174	FALSE	1	107186288	107219320	2840
455.0379669	-0.42759	0.00084898	down	0.14162	FALSE	2	83684329	83689242	4834
133.9074971	-0.43157	0.041988	down	0.78237	FALSE	8	78381994	78387069	3800
183.2920458	-0.43261	0.01864	down	0.63248	FALSE	16	35871799	35946987	6511
3843.019606	-0.43304	5.61E-05	down	0.026375	FALSE	14	88314606	88337467	6425
162.0582141	-0.43783	0.034475	down	0.7376	FALSE	2	68827562	68869884	6837
192.875686	-0.43947	0.026779	down	0.6913	FALSE	14	125405975	126180790	7260
204.5139366	-0.44185	0.028699	down	0.70929	FALSE	9	53079550	53178881	7201
151.9444048	-0.44545	0.037173	down	0.75199	FALSE	1	203936657	204383846	3894
140.8581515	-0.45286	0.029039	down	0.70929	FALSE	7	61826015	61832830	6816
1336.772309	-0.45363	6.09E-05	down	0.026395	FALSE	4	122826992	122837672	1348
128.7010128	-0.45522	0.037102	down	0.75195	FALSE	16	54553933	55220310	7427
124.3124132	-0.46161	0.043023	down	0.78536	FALSE	4	122232587	122365707	5742
141.6800718	-0.46422	0.033738	down	0.7376	FALSE	5	2980069	3204803	12546
294.3317253	-0.46754	0.013505	down	0.55821	FALSE	2	62364501	62402344	8095
558.4699865	-0.46831	0.0036083	down	0.32243	FALSE	7	66915876	67462089	7939
586.0414903	-0.47415	0.00089654	down	0.14174	FALSE	13	65134057	65153489	5652
970.1204899	-0.47793	0.00024041	down	0.065168	FALSE	1	229581650	229848914	1555
149.5245813	-0.47799	0.019062	down	0.63279	FALSE	11	24784407	24971045	5095
235.1137917	-0.47866	0.010415	down	0.5159	FALSE	11	38524034	39454240	4507
101.3867758	-0.4814	0.049647	down	0.82931	FALSE	X	53609176	53805578	4021
136.2107872	-0.48545	0.019343	down	0.63725	FALSE	16	55418397	55595189	3572
420.9846519	-0.49061	0.0031386	down	0.30601	FALSE	17	11008684	11109928	7118
101.8119335	-0.49551	0.044636	down	0.79998	FALSE	12	25492525	25553715	2896
253.0980554	-0.49873	0.0037727	down	0.3299	FALSE	3	40637436	40694818	8026
877.5631703	-0.50321	0.0024832	down	0.27041	FALSE	8	104677580	104873791	5964
16650.28375	-0.50624	1.20E-07	down	0.0003453	FALSE	8	75337133	75345926	2179
115.241621	-0.53074	0.018631	down	0.63248	FALSE	X	7237942	7620011	2911
1846.538776	-0.53644	2.06E-06	down	0.0022317	FALSE	14	109571945	109589160	3439
91.66655475	-0.54175	0.040494	down	0.76899	FALSE	18	10381209	10451255	4362
122.9944829	-0.54407	0.026968	down	0.6913	FALSE	13	124633684	124646648	1950
114.8031838	-0.5457	0.015762	down	0.59614	FALSE	4	88521893	88560382	3120
145.3171554	-0.54724	0.011186	down	0.52941	FALSE	7	59318010	59347267	3031
918.2314007	-0.5494	0.00026275	down	0.066255	FALSE	14	91103518	91138499	3973
110.8700019	-0.55427	0.023337	down	0.67983	FALSE	4	88140953	88471994	6345
468.3773454	-0.55996	1.31E-05	down	0.0088534	FALSE	8	19535483	19649695	3172
1359.261604	-0.57569	5.40E-06	down	0.0048764	FALSE	4	115540950	115840708	7365

93.85706757	-0.578	0.028803	down	0.70929	FALSE	11	3840489	3984898	3323
170.9170108	-0.57959	0.007726	down	0.47598	FALSE	1	145911045	146017701	4412
177.8928307	-0.58267	0.0059994	down	0.40155	FALSE	3	118521296	118932446	9189
159.6717116	-0.58636	0.010443	down	0.5159	FALSE	4	73810188	74134229	4186
85.05954467	-0.58682	0.029807	down	0.70956	FALSE	15	31778593	31790465	5456
89.60332054	-0.59406	0.020637	down	0.64724	FALSE	6	73297158	74402790	2591
103.4556605	-0.59467	0.030506	down	0.71323	FALSE	1	168751423	169323977	4533
131.4784296	-0.59948	0.0088703	down	0.49041	FALSE	8	70788193	70896558	4327
5433.327407	-0.59993	4.36E-07	down	0.00067526	FALSE	15	121427103	121435424	2677
84.15110078	-0.63619	0.032679	down	0.73119	FALSE	3	2814328	3324799	6482
107.0120136	-0.63907	0.01088	down	0.52941	FALSE	17	54371631	54374699	3063
61.00815203	-0.64817	0.042356	down	0.78507	FALSE	14	11300044	11336155	5042
138.3241862	-0.67865	0.0033765	down	0.31232	FALSE	13	89897148	89956222	4347
61.30914715	-0.68586	0.045806	down	0.80993	FALSE	13	5314358	5417591	4284
55.70634847	-0.72247	0.029971	down	0.70956	FALSE	9	39699597	39731036	1395
92.00354479	-0.73293	0.0057489	down	0.40034	FALSE	11	69710364	70022727	6976
466.4251861	-0.74076	3.03E-08	down	0.00016403	FALSE	15	118857479	118878522	6005
59.46639873	-0.77549	0.016097	down	0.60027	FALSE	8	135002320	135021197	3391
748.9540553	-0.79828	1.20E-06	down	0.0014484	FALSE	1	229660101	229666407	4800
119.2913362	-0.8311	0.00068381	down	0.11959	FALSE	6	72103242	72174862	5358
34.25075076	-0.85071	0.049572	down	NA	FALSE	4	40473013	40489032	1501
80.66810524	-0.88034	0.0089133	down	0.49041	FALSE	6	75166990	75174893	5775
48.90477162	-0.89519	0.014027	down	NA	FALSE	X	3891945	4076900	4452
58.18460605	-0.89812	0.0090004	down	0.49041	FALSE	1	120322432	120399041	4336
28.76317616	-0.92991	0.046627	down	NA	FALSE	18	396263	518102	1906
44.66711478	-1.0822	0.0080172	down	NA	TRUE	5	6589864	6662432	2949
26.05335299	-1.1262	0.030555	down	NA	TRUE	1	21059468	21096331	1597
18.91475691	-1.5514	0.014227	down	NA	TRUE	2	69553930	69579089	375
12.90603603	-2.2341	0.027912	down	NA	TRUE	AEMK02000695.1	10196	10348	153
4.523536497	-3.6143	0.033929	down	NA	TRUE	X	59550987	59551071	85

Gene name	Gene description
DYNLRB2	dynein light chain roadblock-type 2 [Source:VGNC Symbol;Acc:VGNC:87502]
SNORD39	Small nucleolar RNA SNORD55/SNORD39 [Source:RFAM;Acc:RF00157]
SPACA1	sperm acrosome associated 1 [Source:VGNC Symbol;Acc:VGNC:93367]
-	phosphatidylinositol 3,4,5-trisphosphate 3-phosphatase TPTE2 [Source:NCBI gene (formerly Entrezgene);Acc:100625564]
-	-
LPAR5	lysophosphatidic acid receptor 5 [Source:VGNC Symbol;Acc:VGNC:89787]
-	-
-	C-C motif chemokine ligand 20 [Source:VGNC Symbol;Acc:VGNC:95484]
-	MRN complex interacting protein [Source:VGNC Symbol;Acc:VGNC:90345]
-	-
HPGD	15-hydroxyprostaglandin dehydrogenase [Source:VGNC Symbol;Acc:VGNC:88961]
-	-
WNT11	Wnt family member 11 [Source:VGNC Symbol;Acc:VGNC:94966]
-	calcitonin receptor-stimulating peptide-2 [Source:NCBI gene (formerly Entrezgene);Acc:396574]
-	-
-	transmembrane protein 234 [Source:VGNC Symbol;Acc:VGNC:94148]
CBLN4	cerebellin 4 precursor [Source:VGNC Symbol;Acc:VGNC:95793]
-	-
-	-
-	-
PIK3IP1	phosphoinositide-3-kinase interacting protein 1 [Source:VGNC Symbol;Acc:VGNC:91444]
-	-
CXCL2	chemokine (C-X-C motif) ligand 2 [Source:NCBI gene (formerly Entrezgene);Acc:414904]
TMEM238	transmembrane protein 238 [Source:VGNC Symbol;Acc:VGNC:94149]
TMEM190	transmembrane protein 190 [Source:VGNC Symbol;Acc:VGNC:94125]
BDNF	brain derived neurotrophic factor [Source:VGNC Symbol;Acc:VGNC:85795]
CXCL8	C-X-C motif chemokine ligand 8 [Source:VGNC Symbol;Acc:VGNC:87106]
HGF	hepatocyte growth factor [Source:VGNC Symbol;Acc:VGNC:88869]
AMCF-II	alveolar macrophage-derived chemotactic factor-II [Source:NCBI gene (formerly Entrezgene);Acc:396900]
IL11	interleukin 11 [Source:VGNC Symbol;Acc:VGNC:89078]
STC1	stanniocalcin 1 [Source:VGNC Symbol;Acc:VGNC:93542]
FOS	Fos proto-oncogene, AP-1 transcription factor subunit [Source:NCBI gene (formerly Entrezgene);Acc:100144486]
SST	somatostatin [Source:VGNC Symbol;Acc:VGNC:93495]
IL6	interleukin 6 [Source:NCBI gene (formerly Entrezgene);Acc:399500]
B4GALT6	beta-1,4-galactosyltransferase 6 [Source:VGNC Symbol;Acc:VGNC:85734]
IL1A	interleukin 1 alpha [Source:VGNC Symbol;Acc:VGNC:89091]

MAMDC2	MAM domain containing 2 [Source:VGNC Symbol;Acc:VGNC:89962]
CXCL14	C-X-C motif chemokine ligand 14 [Source:VGNC Symbol;Acc:VGNC:87105]
-	-
CDA	cytidine deaminase [Source:VGNC Symbol;Acc:VGNC:86439]
SERPINB2	serpin family B member 2 [Source:VGNC Symbol;Acc:VGNC:92737]
-	-
-	-
FBXL15	F-box and leucine rich repeat protein 15 [Source:VGNC Symbol;Acc:VGNC:98002]
PTGS2	prostaglandin-endoperoxide synthase 2 [Source:VGNC Symbol;Acc:VGNC:91959]
ANGPT1	angiopoietin 1 [Source:VGNC Symbol;Acc:VGNC:98732]
DUSP4	dual specificity phosphatase 4 [Source:VGNC Symbol;Acc:VGNC:96248]
-	family with sequence similarity 71 member E2 [Source:NCBI gene (formerly Entrezgene);Acc:110261376]
LRATD1	LRAT domain containing 1 [Source:VGNC Symbol;Acc:VGNC:89799]
POPDC3	popeye domain containing 3 [Source:VGNC Symbol;Acc:VGNC:91668]
-	-
ECEL1	endothelin converting enzyme like 1 [Source:VGNC Symbol;Acc:VGNC:96259]
TGM2	transglutaminase 2 [Source:VGNC Symbol;Acc:VGNC:95542]
-	-
LRRC31	leucine rich repeat containing 31 [Source:VGNC Symbol;Acc:VGNC:89834]
PTPRN	protein tyrosine phosphatase receptor type N [Source:VGNC Symbol;Acc:VGNC:96523]
NR4A3	nuclear receptor subfamily 4 group A member 3 [Source:VGNC Symbol;Acc:VGNC:90885]
FGL2	fibrinogen like 2 [Source:VGNC Symbol;Acc:VGNC:88123]
-	-
RASL11A	RAS like family 11 member A [Source:VGNC Symbol;Acc:VGNC:92119]
C1orf122	chromosome 6 C1orf122 homolog [Source:VGNC Symbol;Acc:VGNC:86060]
EDNRB	endothelin receptor type B [Source:VGNC Symbol;Acc:VGNC:87550]
HS3ST3B1	heparan sulfate-glucosamine 3-sulfotransferase 3B1 [Source:VGNC Symbol;Acc:VGNC:99003]
SLC2A5	solute carrier family 2 member 5 [Source:VGNC Symbol;Acc:VGNC:93050]
RHBDD3	rhomboid domain containing 3 [Source:VGNC Symbol;Acc:VGNC:98290]
DRD1	dopamine receptor D1 [Source:VGNC Symbol;Acc:VGNC:87443]
SLC16A3	solute carrier family 16 member 3 [Source:VGNC Symbol;Acc:VGNC:92943]
VCAM1	vascular cell adhesion molecule 1 [Source:VGNC Symbol;Acc:VGNC:94811]
LMO2	LIM domain only 2 [Source:VGNC Symbol;Acc:VGNC:89765]
-	DNA methyltransferase 3 like [Source:NCBI gene (formerly Entrezgene);Acc:100621554]
PLEKHH3	pleckstrin homology, MyTH4 and FERM domain containing H3 [Source:VGNC Symbol;Acc:VGNC:91550]
CTSL	cathepsin L [Source:NCBI gene (formerly Entrezgene);Acc:100515919]
CD96	CD96 molecule [Source:VGNC Symbol;Acc:VGNC:86437]

APLN	apelin [Source:NCBI gene (formerly Entrezgene);Acc:100625006]
SPRY2	sprouty RTK signaling antagonist 2 [Source:VGNC Symbol;Acc:VGNC:93425]
IL17D	interleukin 17D [Source:NCBI gene (formerly Entrezgene);Acc:100738902]
TEDC2	tubulin epsilon and delta complex 2 [Source:VGNC Symbol;Acc:VGNC:93862]
-	-
SAPCD2	suppressor APC domain containing 2 [Source:VGNC Symbol;Acc:VGNC:92578]
AREG	amphiregulin [Source:VGNC Symbol;Acc:VGNC:85444]
NR4A2	nuclear receptor subfamily 4 group A member 2 [Source:VGNC Symbol;Acc:VGNC:96451]
RNF144B	ring finger protein 144B [Source:VGNC Symbol;Acc:VGNC:92359]
RIPOR2	RHO family interacting cell polarization regulator 2 [Source:VGNC Symbol;Acc:VGNC:92322]
CIART	circadian associated repressor of transcription [Source:VGNC Symbol;Acc:VGNC:86694]
EXOSC4	exosome component 4 [Source:VGNC Symbol;Acc:VGNC:87840]
-	biogenesis of lysosomal organelles complex 1 subunit 1 [Source:NCBI gene (formerly Entrezgene);Acc:100517399]
PTGES	prostaglandin E synthase [Source:VGNC Symbol;Acc:VGNC:91950]
-	-
PWWP3B	PWWP domain containing 3B [Source:VGNC Symbol;Acc:VGNC:92012]
NDUFA4L2	NDUFA4 mitochondrial complex associated like 2 [Source:VGNC Symbol;Acc:VGNC:90636]
HOXC5	homeobox C5 [Source:NCBI gene (formerly Entrezgene);Acc:100152707]
IGSF8	immunoglobulin superfamily member 8 [Source:VGNC Symbol;Acc:VGNC:89069]
CCL2	chemokine (C-C motif) ligand 2 [Source:NCBI gene (formerly Entrezgene);Acc:397422]
CCNG2	cyclin G2 [Source:VGNC Symbol;Acc:VGNC:86358]
RGS5	regulator of G protein signaling 5 [Source:VGNC Symbol;Acc:VGNC:98844]
PI15	peptidase inhibitor 15 [Source:VGNC Symbol;Acc:VGNC:98188]
ADAMTS5	ADAM metallopeptidase with thrombospondin type 1 motif 5 [Source:VGNC Symbol;Acc:VGNC:85085]
TRIM39	tripartite motif containing 39 [Source:NCBI gene (formerly Entrezgene);Acc:100151742]
NR4A1	nuclear receptor subfamily 4 group A member 1 [Source:HGNC Symbol;Acc:HGNC:7980]
AWN	sperm associated AWN protein [Source:NCBI gene (formerly Entrezgene);Acc:396783]
SIVA1	SIVA1 apoptosis inducing factor [Source:NCBI gene (formerly Entrezgene);Acc:110258343]
-	salt inducible kinase 1 [Source:NCBI gene (formerly Entrezgene);Acc:100621265]
UMAD1	UBAP1-MVB12-associated (UMA) domain containing 1 [Source:NCBI gene (formerly Entrezgene);Acc:102165755]
SEMA3A	semaphorin 3A [Source:VGNC Symbol;Acc:VGNC:92693]
-	phosphatidylethanolamine N-methyltransferase [Source:VGNC Symbol;Acc:VGNC:91311]
-	succinate dehydrogenase complex assembly factor 3 [Source:NCBI gene (formerly Entrezgene);Acc:102158000]
PDK4	pyruvate dehydrogenase kinase 4 [Source:VGNC Symbol;Acc:VGNC:91280]
BNIP3	BCL2 interacting protein 3 [Source:VGNC Symbol;Acc:VGNC:85855]
FGF2	fibroblast growth factor 2 [Source:VGNC Symbol;Acc:VGNC:98928]
ARL6IP4	ADP ribosylation factor like GTPase 6 interacting protein 4 [Source:NCBI gene (formerly Entrezgene);Acc:100157752]

CENPW	centromere protein W [Source:NCBI gene (formerly Entrezgene);Acc:100513385]
USP35	ubiquitin specific peptidase 35 [Source:VGNC Symbol;Acc:VGNC:94760]
ESM1	endothelial cell specific molecule 1 [Source:VGNC Symbol;Acc:VGNC:87789]
CCDC160	coiled-coil domain containing 160 [Source:VGNC Symbol;Acc:VGNC:86269]
-	-
-	stromal cell derived factor 2 like 1 [Source:NCBI gene (formerly Entrezgene);Acc:100156780]
PLA2G4A	phospholipase A2 group IVA [Source:VGNC Symbol;Acc:VGNC:91495]
TROAP	trophinin associated protein [Source:VGNC Symbol;Acc:VGNC:94458]
ADAMTS1	ADAM metallopeptidase with thrombospondin type 1 motif 1 [Source:VGNC Symbol;Acc:VGNC:85072]
CTSC	cathepsin C [Source:VGNC Symbol;Acc:VGNC:87075]
CDC14A	cell division cycle 14A [Source:VGNC Symbol;Acc:VGNC:86442]
-	uncharacterized LOC110259374 [Source:NCBI gene (formerly Entrezgene);Acc:110259374]
LIF	LIF interleukin 6 family cytokine [Source:VGNC Symbol;Acc:VGNC:89719]
RPA3	replication protein A3 [Source:VGNC Symbol;Acc:VGNC:92415]
-	-
PENK	proenkephalin [Source:VGNC Symbol;Acc:VGNC:91312]
PRR12	proline rich 12 [Source:VGNC Symbol;Acc:VGNC:91851]
ID2	inhibitor of DNA binding 2 [Source:VGNC Symbol;Acc:VGNC:98041]
LVRN	laeverin [Source:VGNC Symbol;Acc:VGNC:89898]
-	-
NFATC2	nuclear factor of activated T cells 2 [Source:VGNC Symbol;Acc:VGNC:96440]
MAFF	MAF bZIP transcription factor F [Source:VGNC Symbol;Acc:VGNC:89947]
-	-
USE1	unconventional SNARE in the ER 1 [Source:VGNC Symbol;Acc:VGNC:94737]
FJX1	four-jointed box kinase 1 [Source:VGNC Symbol;Acc:VGNC:88142]
HMGA1	high mobility group AT-hook 1 [Source:NCBI gene (formerly Entrezgene);Acc:100157848]
YAF2	YY1 associated factor 2 [Source:VGNC Symbol;Acc:VGNC:95018]
FAM110B	family with sequence similarity 110 member B [Source:VGNC Symbol;Acc:VGNC:87885]
KLHL26	kelch like family member 26 [Source:VGNC Symbol;Acc:VGNC:89522]
RASD1	ras related dexamethasone induced 1 [Source:VGNC Symbol;Acc:VGNC:92107]
-	-
METTL26	methyltransferase like 26 [Source:VGNC Symbol;Acc:VGNC:90163]
PKIG	cAMP-dependent protein kinase inhibitor gamma [Source:VGNC Symbol;Acc:VGNC:96486]
PSMG3	proteasome assembly chaperone 3 [Source:VGNC Symbol;Acc:VGNC:91929]
ZNRF2	zinc and ring finger 2 [Source:VGNC Symbol;Acc:VGNC:95317]
PNRC1	proline rich nuclear receptor coactivator 1 [Source:VGNC Symbol;Acc:VGNC:91613]
CISD2	CDGSH iron sulfur domain 2 [Source:VGNC Symbol;Acc:VGNC:86707]

MMP9	matrix metallopeptidase 9 [Source:VGNC Symbol;Acc:VGNC:96422]
LENG8	leukocyte receptor cluster member 8 [Source:VGNC Symbol;Acc:VGNC:89681]
RGS2	regulator of G protein signaling 2 [Source:VGNC Symbol;Acc:VGNC:96538]
-	centromere protein X [Source:NCBI gene (formerly Entrezgene);Acc:110255926]
SELENOW	selenoprotein W [Source:NCBI gene (formerly Entrezgene);Acc:397032]
AHR	aryl hydrocarbon receptor [Source:NCBI gene (formerly Entrezgene);Acc:396654]
SYNGAP1	synaptic Ras GTPase activating protein 1 [Source:VGNC Symbol;Acc:VGNC:93664]
IGFBP2	insulin like growth factor binding protein 2 [Source:NCBI gene (formerly Entrezgene);Acc:397064]
CORO2B	coronin 2B [Source:VGNC Symbol;Acc:VGNC:86916]
TIMP1	TIMP metallopeptidase inhibitor 1 [Source:NCBI gene (formerly Entrezgene);Acc:396862]
-	-
MMD	monocyte to macrophage differentiation associated [Source:VGNC Symbol;Acc:VGNC:90264]
-	-
-	angiotensin II receptor associated protein [Source:VGNC Symbol;Acc:VGNC:85193]
MAMDC4	14 kDa phosphohistidine phosphatase [Source:NCBI gene (formerly Entrezgene);Acc:100513261]
ABTB1	ankyrin repeat and BTB domain containing 1 [Source:VGNC Symbol;Acc:VGNC:84993]
IER3	immediate early response 3 [Source:VGNC Symbol;Acc:VGNC:89027]
ABHD6	abhydrolase domain containing 6, acylglycerol lipase [Source:VGNC Symbol;Acc:VGNC:84982]
-	mitochondrial fission process 1 [Source:VGNC Symbol;Acc:VGNC:90446]
PDXK	pyridoxal kinase [Source:NCBI gene (formerly Entrezgene);Acc:396983]
PRDX6	peroxiredoxin 6 [Source:VGNC Symbol;Acc:VGNC:91785]
MRPL20	mitochondrial ribosomal protein L20 [Source:VGNC Symbol;Acc:VGNC:90356]
LPL	lipoprotein lipase [Source:VGNC Symbol;Acc:VGNC:89795]
PRELP	proline and arginine rich end leucine rich repeat protein [Source:VGNC Symbol;Acc:VGNC:91789]
ADAMTS7	ADAM metallopeptidase with thrombospondin type 1 motif 7 [Source:VGNC Symbol;Acc:VGNC:85087]
APIP	APAF1 interacting protein [Source:VGNC Symbol;Acc:VGNC:85411]
SLC9A3R2	SLC9A3 regulator 2 [Source:VGNC Symbol;Acc:VGNC:93185]
ARSL	arylsulfatase E (chondrodysplasia punctata 1) [Source:NCBI gene (formerly Entrezgene);Acc:100627778]
PFKFB3	6-phosphofructo-2-kinase/fructose-2,6-biphosphatase 3 [Source:VGNC Symbol;Acc:VGNC:96480]
-	DNA polymerase alpha 2, accessory subunit [Source:NCBI gene (formerly Entrezgene);Acc:100513971]
CTU2	cytosolic thiouridylase subunit 2 [Source:VGNC Symbol;Acc:VGNC:87083]
HBEGF	heparin binding EGF like growth factor [Source:VGNC Symbol;Acc:VGNC:88792]
TBX2	T-box transcription factor 2 [Source:VGNC Symbol;Acc:VGNC:93798]
-	-
ENOSF1	enolase superfamily member 1 [Source:VGNC Symbol;Acc:VGNC:87705]
SNX16	sorting nexin 16 [Source:VGNC Symbol;Acc:VGNC:93308]
HOXD8	homeobox D8 [Source:VGNC Symbol;Acc:VGNC:96356]

ID1	inhibitor of DNA binding 1, HLH protein [Source:VGNC Symbol;Acc:VGNC:96366]
CCDC8	coiled-coil domain containing 8 [Source:VGNC Symbol;Acc:VGNC:86314]
EDNRA	endothelin receptor type A [Source:VGNC Symbol;Acc:VGNC:87549]
-	-
-	NADH:ubiquinone oxidoreductase subunit V3 [Source:VGNC Symbol;Acc:VGNC:90658]
ERRFI1	ERBB receptor feedback inhibitor 1 [Source:VGNC Symbol;Acc:VGNC:87784]
-	glycerol kinase [Source:NCBI gene (formerly Entrezgene);Acc:100233182]
IGFBP4	insulin like growth factor binding protein 4 [Source:VGNC Symbol;Acc:VGNC:98045]
TFPT	TCF3 fusion partner [Source:VGNC Symbol;Acc:VGNC:93923]
-	arginine-glutamic acid dipeptide repeats [Source:VGNC Symbol;Acc:VGNC:92216]
-	BLOC-1 related complex subunit 7 [Source:NCBI gene (formerly Entrezgene);Acc:100157047]
RASL11B	RAS like family 11 member B [Source:VGNC Symbol;Acc:VGNC:92120]
-	-
GALK2	fibroblast growth factor 7 [Source:NCBI gene (formerly Entrezgene);Acc:397281]
-	-
FLOT1	flotillin 1 [Source:VGNC Symbol;Acc:VGNC:88158]
SAT1	spermidine/spermine N1-acetyltransferase 1 [Source:NCBI gene (formerly Entrezgene);Acc:397645]
CREM	cAMP responsive element modulator [Source:VGNC Symbol;Acc:VGNC:96010]
CYB561	cytochrome b561 [Source:VGNC Symbol;Acc:VGNC:87119]
PDE4D	phosphodiesterase 4D [Source:VGNC Symbol;Acc:VGNC:91256]
-	-
-	-
-	malignant T-cell-amplified sequence 1 [Source:NCBI gene (formerly Entrezgene);Acc:100738684]
MRPS26	mitochondrial ribosomal protein S26 [Source:VGNC Symbol;Acc:VGNC:95601]
PDE4B	phosphodiesterase 4B [Source:VGNC Symbol;Acc:VGNC:91255]
HTR2A	5-hydroxytryptamine receptor 2A [Source:NCBI gene (formerly Entrezgene);Acc:397432]
-	inducible T cell costimulator ligand [Source:NCBI gene (formerly Entrezgene);Acc:100621467]
SLC43A2	solute carrier family 43 member 2 [Source:VGNC Symbol;Acc:VGNC:93117]
-	-
ADM	adrenomedullin [Source:VGNC Symbol;Acc:VGNC:85144]
BLOC1S2	biogenesis of lysosomal organelles complex 1 subunit 2 [Source:VGNC Symbol;Acc:VGNC:85830]
-	jumping translocation breakpoint [Source:NCBI gene (formerly Entrezgene);Acc:100145894]
WFDC1	WAP four-disulfide core domain 1 [Source:VGNC Symbol;Acc:VGNC:94953]
CFLAR	CASP8 and FADD like apoptosis regulator [Source:VGNC Symbol;Acc:VGNC:95894]
SURF2	surfeit 2 [Source:VGNC Symbol;Acc:VGNC:93630]
TPBG	trophoblast glycoprotein [Source:VGNC Symbol;Acc:VGNC:94332]
-	dihydrofolate reductase [Source:NCBI gene (formerly Entrezgene);Acc:100525912]

MASP1	mannan binding lectin serine peptidase 1 [Source:VGNC Symbol;Acc:VGNC:90032]
TLE5	TLE family member 5, transcriptional modulator [Source:NCBI gene (formerly Entrezgene);Acc:110259367]
ALAD	aminolevulinate dehydratase [Source:VGNC Symbol;Acc:VGNC:85234]
-	prothymosin alpha [Source:NCBI gene (formerly Entrezgene);Acc:100294684]
MED28	mediator complex subunit 28 [Source:VGNC Symbol;Acc:VGNC:90118]
ASF1B	anti-silencing function 1B histone chaperone [Source:VGNC Symbol;Acc:VGNC:85574]
TNFAIP6	TNF alpha induced protein 6 [Source:VGNC Symbol;Acc:VGNC:95544]
DRAM1	DNA damage regulated autophagy modulator 1 [Source:VGNC Symbol;Acc:VGNC:87436]
EMC10	ER membrane protein complex subunit 10 [Source:VGNC Symbol;Acc:VGNC:87669]
JDP2	Jun dimerization protein 2 [Source:VGNC Symbol;Acc:VGNC:89282]
HES1	hes family bHLH transcription factor 1 [Source:VGNC Symbol;Acc:VGNC:88854]
ZFP36L1	ZFP36 ring finger protein like 1 [Source:VGNC Symbol;Acc:VGNC:95146]
BTF3L4	basic transcription factor 3 like 4 [Source:VGNC Symbol;Acc:VGNC:85912]
MARCHF3	membrane associated ring-CH-type finger 3 [Source:VGNC Symbol;Acc:VGNC:90020]
PMAIP1	phorbol-12-myristate-13-acetate-induced protein 1 [Source:NCBI gene (formerly Entrezgene);Acc:397278]
GASK1B	golgi associated kinase 1B [Source:VGNC Symbol;Acc:VGNC:88362]
MED6	mediator complex subunit 6 [Source:VGNC Symbol;Acc:VGNC:90122]
NIP7	nucleolar pre-rRNA processing protein NIP7 [Source:VGNC Symbol;Acc:VGNC:90747]
KIFC3	kinesin family member C3 [Source:VGNC Symbol;Acc:VGNC:89479]
HUWE1	HECT, UBA and WWE domain containing E3 ubiquitin protein ligase 1 [Source:VGNC Symbol;Acc:VGNC:89008]
FOSL2	FOS like 2, AP-1 transcription factor subunit [Source:VGNC Symbol;Acc:VGNC:88192]
REV3L	REV3 like, DNA directed polymerase zeta catalytic subunit [Source:VGNC Symbol;Acc:VGNC:92226]
TLN2	talin 2 [Source:NCBI gene (formerly Entrezgene);Acc:100156660]
WWP1	WW domain containing E3 ubiquitin protein ligase 1 [Source:VGNC Symbol;Acc:VGNC:94989]
MAN2A2	mannosidase alpha class 2A member 2 [Source:VGNC Symbol;Acc:VGNC:98099]
MYO9A	myosin IXA [Source:NCBI gene (formerly Entrezgene);Acc:100157683]
FBLN2	fibulin 2 [Source:NCBI gene (formerly Entrezgene);Acc:100621722]
SPARC	secreted protein acidic and cysteine rich [Source:VGNC Symbol;Acc:VGNC:98332]
-	lysine methyltransferase 2A [Source:NCBI gene (formerly Entrezgene);Acc:100623006]
ZNFX1	zinc finger NFX1-type containing 1 [Source:VGNC Symbol;Acc:VGNC:96210]
RANBP17	RAN binding protein 17 [Source:VGNC Symbol;Acc:VGNC:92078]
ARNT2	aryl hydrocarbon receptor nuclear translocator 2 [Source:NCBI gene (formerly Entrezgene);Acc:100157219]
TRIO	trio Rho guanine nucleotide exchange factor [Source:VGNC Symbol;Acc:VGNC:94435]
LPP	LIM domain containing preferred translocation partner in lipoma [Source:NCBI gene (formerly Entrezgene);Acc:100626969]
ARHGEF2	Rho/Rac guanine nucleotide exchange factor 2 [Source:VGNC Symbol;Acc:VGNC:85493]
TRIB3	tribbles pseudokinase 3 [Source:VGNC Symbol;Acc:VGNC:95791]
CCN4	cellular communication network factor 4 [Source:VGNC Symbol;Acc:VGNC:86346]

CIPC	CLOCK interacting pacemaker [Source:VGNC Symbol;Acc:VGNC:97929]
NIBAN1	niban apoptosis regulator 1 [Source:VGNC Symbol;Acc:VGNC:90740]
UTRN	utrophin [Source:NCBI gene (formerly Entrezgene);Acc:100523292]
ROR2	receptor tyrosine kinase like orphan receptor 2 [Source:VGNC Symbol;Acc:VGNC:92407]
-	T-box transcription factor 15 [Source:VGNC Symbol;Acc:VGNC:93796]
JADE1	jade family PHD finger 1 [Source:VGNC Symbol;Acc:VGNC:89266]
FLNC	filamin C [Source:VGNC Symbol;Acc:VGNC:88157]
ADAMTS6	ADAM metallopeptidase with thrombospondin type 1 motif 6 [Source:VGNC Symbol;Acc:VGNC:85086]
STAT3	signal transducer and activator of transcription 3 [Source:VGNC Symbol;Acc:VGNC:93540]
RASA3	RAS p21 protein activator 3 [Source:VGNC Symbol;Acc:VGNC:92103]
-	myosin heavy chain 10 [Source:NCBI gene (formerly Entrezgene);Acc:396903]
LRRK1	leucine rich repeat kinase 1 [Source:VGNC Symbol;Acc:VGNC:98093]
DAAM1	dishevelled associated activator of morphogenesis 1 [Source:VGNC Symbol;Acc:VGNC:87141]
KCNAB2	potassium voltage-gated channel subfamily A regulatory beta subunit 2 [Source:VGNC Symbol;Acc:VGNC:89329]
CHUK	component of inhibitor of nuclear factor kappa B kinase complex [Source:VGNC Symbol;Acc:VGNC:86688]
SH2B3	SH2B adaptor protein 3 [Source:VGNC Symbol;Acc:VGNC:92807]
LIPG	lipase G, endothelial type [Source:VGNC Symbol;Acc:VGNC:89739]
NCOA5	nuclear receptor coactivator 5 [Source:VGNC Symbol;Acc:VGNC:95702]
DUSP14	dual specificity phosphatase 14 [Source:VGNC Symbol;Acc:VGNC:87479]
APC	APC regulator of WNT signaling pathway [Source:VGNC Symbol;Acc:VGNC:99584]
RASL12	RAS like family 12 [Source:VGNC Symbol;Acc:VGNC:92121]
SYNC	syncoilin, intermediate filament protein [Source:VGNC Symbol;Acc:VGNC:93660]
-	transforming acidic coiled-coil containing protein 1 [Source:NCBI gene (formerly Entrezgene);Acc:100624903]
UBR2	ubiquitin protein ligase E3 component n-recognin 2 [Source:VGNC Symbol;Acc:VGNC:94669]
-	myosin heavy chain 9 [Source:VGNC Symbol;Acc:VGNC:90512]
FAM78B	family with sequence similarity 78 member B [Source:VGNC Symbol;Acc:VGNC:87985]
TNFAIP1	TNF alpha induced protein 1 [Source:VGNC Symbol;Acc:VGNC:94250]
SCRN1	secernin 1 [Source:VGNC Symbol;Acc:VGNC:92647]
SMG1	SMG1 nonsense mediated mRNA decay associated PI3K related kinase [Source:VGNC Symbol;Acc:VGNC:93248]
TTC28	tetratricopeptide repeat domain 28 [Source:VGNC Symbol;Acc:VGNC:94543]
RHOU	ras homolog family member U [Source:VGNC Symbol;Acc:VGNC:92294]
GTPBP2	GTP binding protein 2 [Source:VGNC Symbol;Acc:VGNC:88743]
CEMIP	cell migration inducing hyaluronidase 1 [Source:VGNC Symbol;Acc:VGNC:86542]
SEMA3D	semaphorin 3D [Source:VGNC Symbol;Acc:VGNC:92696]
DOCK9	dedicator of cytokinesis 9 [Source:VGNC Symbol;Acc:VGNC:87399]
TAOK3	TAO kinase 3 [Source:VGNC Symbol;Acc:VGNC:98357]
STK10	serine/threonine kinase 10 [Source:NCBI gene (formerly Entrezgene);Acc:100524465]

NOD1	nucleotide binding oligomerization domain containing 1 [Source:NCBI gene (formerly Entrezgene);Acc:100135660]
CCDC80	coiled-coil domain containing 80 [Source:VGNC Symbol;Acc:VGNC:86315]
ARHGAP21	Rho GTPase activating protein 21 [Source:VGNC Symbol;Acc:VGNC:96018]
IFFO2	intermediate filament family orphan 2 [Source:VGNC Symbol;Acc:VGNC:98475]
-	BRCA2 DNA repair associated [Source:NCBI gene (formerly Entrezgene);Acc:100624979]
PCSK6	proprotein convertase subtilisin/kexin type 6 [Source:NCBI gene (formerly Entrezgene);Acc:100524545]
ALMS1	ALMS1 centrosome and basal body associated protein [Source:VGNC Symbol;Acc:VGNC:85264]
MYO9B	myosin IXB [Source:VGNC Symbol;Acc:VGNC:90534]
SYNE2	spectrin repeat containing nuclear envelope protein 2 [Source:VGNC Symbol;Acc:VGNC:93662]
-	-
GOLGB1	golgin B1 [Source:VGNC Symbol;Acc:VGNC:88554]
FRYL	FRY like transcription coactivator [Source:VGNC Symbol;Acc:VGNC:98015]
ROBO1	roundabout guidance receptor 1 [Source:NCBI gene (formerly Entrezgene);Acc:100517310]
ELN	elastin [Source:VGNC Symbol;Acc:VGNC:87655]
RPS6KA2	ribosomal protein S6 kinase A2 [Source:VGNC Symbol;Acc:VGNC:92441]
SCD	stearoyl-CoA desaturase [Source:NCBI gene (formerly Entrezgene);Acc:396670]
TP53BP1	tumor protein p53 binding protein 1 [Source:VGNC Symbol;Acc:VGNC:94325]
TET2	tet methylcytosine dioxygenase 2 [Source:VGNC Symbol;Acc:VGNC:93889]
-	FRY microtubule binding protein [Source:NCBI gene (formerly Entrezgene);Acc:100155799]
MAGI1	membrane associated guanylate kinase, WW and PDZ domain containing 1 [Source:VGNC Symbol;Acc:VGNC:98097]
SETX	senataxin [Source:VGNC Symbol;Acc:VGNC:92766]
ADCY7	adenylate cyclase 7 [Source:VGNC Symbol;Acc:VGNC:85111]
PLXNA4	plexin A4 [Source:VGNC Symbol;Acc:VGNC:98204]
NINL	ninein like [Source:VGNC Symbol;Acc:VGNC:96204]
PEX1	peroxisomal biogenesis factor 1 [Source:VGNC Symbol;Acc:VGNC:91319]
EML1	EMAP like 1 [Source:VGNC Symbol;Acc:VGNC:87682]
ZNF329	zinc finger protein 329 [Source:VGNC Symbol;Acc:VGNC:95225]
TBL1XR1	TBL1X receptor 1 [Source:VGNC Symbol;Acc:VGNC:96600]
MEGF6	multiple EGF like domains 6 [Source:VGNC Symbol;Acc:VGNC:98499]
OSBPL10	oxysterol binding protein like 10 [Source:VGNC Symbol;Acc:VGNC:91068]
MTF1	metal regulatory transcription factor 1 [Source:VGNC Symbol;Acc:VGNC:90443]
SNX30	sorting nexin family member 30 [Source:VGNC Symbol;Acc:VGNC:93318]
PHACTR1	phosphatase and actin regulator 1 [Source:VGNC Symbol;Acc:VGNC:91365]
KAT6B	lysine acetyltransferase 6B [Source:VGNC Symbol;Acc:VGNC:89306]
DCHS2	dachsous cadherin-related 2 [Source:NCBI gene (formerly Entrezgene);Acc:100626442]
LCOR	ligand-dependent corepressor [Source:NCBI gene (formerly Entrezgene);Acc:100627422]
MYCBP2	MYC binding protein 2 [Source:VGNC Symbol;Acc:VGNC:90502]

SSH1	slingshot protein phosphatase 1 [Source:VGNC Symbol;Acc:VGNC:93485]
TMEM35A	transmembrane protein 35A [Source:VGNC Symbol;Acc:VGNC:94172]
-	DEP domain containing 5, GATOR1 subcomplex subunit [Source:NCBI gene (formerly Entrezgene);Acc:100156710]
PECAM1	platelet and endothelial cell adhesion molecule 1 [Source:VGNC Symbol;Acc:VGNC:91304]
CNN1	calponin 1 [Source:VGNC Symbol;Acc:VGNC:86825]
SOCS7	suppressor of cytokine signaling 7 [Source:VGNC Symbol;Acc:VGNC:93334]
LIMS2	LIM zinc finger domain containing 2 [Source:VGNC Symbol;Acc:VGNC:95731]
ITPR1	inositol 1,4,5-trisphosphate receptor type 1 [Source:VGNC Symbol;Acc:VGNC:89253]
USP53	ubiquitin specific peptidase 53 [Source:VGNC Symbol;Acc:VGNC:94775]
PRKCA	protein kinase C alpha [Source:VGNC Symbol;Acc:VGNC:99028]
GOLGA4	golgin A4 [Source:VGNC Symbol;Acc:VGNC:88551]
RCAN2	regulator of calcineurin 2 [Source:VGNC Symbol;Acc:VGNC:92171]
YEATS2	YEATS domain containing 2 [Source:VGNC Symbol;Acc:VGNC:95023]
ADD2	adducin 2 [Source:VGNC Symbol;Acc:VGNC:85117]
EPHB3	EPH receptor B3 [Source:VGNC Symbol;Acc:VGNC:87738]
ITGA8	integrin subunit alpha 8 [Source:VGNC Symbol;Acc:VGNC:96379]
-	-
HMCN1	hemicentin 1 [Source:NCBI gene (formerly Entrezgene);Acc:102166693]
CRYBG3	crystallin beta-gamma domain containing 3 [Source:VGNC Symbol;Acc:VGNC:87020]
CADPS2	calcium dependent secretion activator 2 [Source:VGNC Symbol;Acc:VGNC:86137]
KIF1A	kinesin family member 1A [Source:NCBI gene (formerly Entrezgene);Acc:100517246]
ZBTB7C	zinc finger and BTB domain containing 7C [Source:VGNC Symbol;Acc:VGNC:95083]
FHOD3	formin homology 2 domain containing 3 [Source:VGNC Symbol;Acc:VGNC:88132]
-	PDZ and LIM domain 2 [Source:VGNC Symbol;Acc:VGNC:91282]
FAM13C	family with sequence similarity 13 member C [Source:VGNC Symbol;Acc:VGNC:87907]
ARSJ	arylsulfatase family member J [Source:VGNC Symbol;Acc:VGNC:85549]
-	-
ACACA	acetyl-CoA carboxylase alpha [Source:VGNC Symbol;Acc:VGNC:84997]
-	-
PCDH7	protocadherin 7 [Source:NCBI gene (formerly Entrezgene);Acc:100520035]
-	zinc finger protein 397 [Source:NCBI gene (formerly Entrezgene);Acc:102164512]
SLCO3A1	solute carrier organic anion transporter family member 3A1 [Source:VGNC Symbol;Acc:VGNC:93197]
-	-
UHRF1BP1	UHRF1 binding protein 1 [Source:VGNC Symbol;Acc:VGNC:94690]
TMEM132E	transmembrane protein 132E [Source:VGNC Symbol;Acc:VGNC:94083]
-	ankyrin 2 [Source:NCBI gene (formerly Entrezgene);Acc:100512728]
FNIP2	folliculin interacting protein 2 [Source:VGNC Symbol;Acc:VGNC:88188]

-	pleckstrin homology domain containing O2 [Source:NCBI gene (formerly Entrezgene);Acc:100154769]
ENC1	ectodermal-neural cortex 1 [Source:VGNC Symbol;Acc:VGNC:87696]
MAB21L2	mab-21 like 2 [Source:VGNC Symbol;Acc:VGNC:89933]
MAP3K1	mitogen-activated protein kinase kinase kinase 1 [Source:VGNC Symbol;Acc:VGNC:98104]
-	annexin A8 [Source:NCBI gene (formerly Entrezgene);Acc:100155930]
COL5A3	collagen type V alpha 3 chain [Source:VGNC Symbol;Acc:VGNC:86878]
ATRNL1	attractin like 1 [Source:VGNC Symbol;Acc:VGNC:85685]
CDON	cell adhesion associated, oncogene regulated [Source:VGNC Symbol;Acc:VGNC:86519]
ADAMTSL1	ADAMTS like 1 [Source:VGNC Symbol;Acc:VGNC:85090]
CLEC14A	C-type lectin domain containing 14A [Source:VGNC Symbol;Acc:VGNC:86747]
F3	coagulation factor III, tissue factor [Source:VGNC Symbol;Acc:VGNC:87864]
SLIT3	slit guidance ligand 3 [Source:VGNC Symbol;Acc:VGNC:93205]
TLCD4	TLC domain containing 4 [Source:VGNC Symbol;Acc:VGNC:98371]
CELSR1	cadherin EGF LAG seven-pass G-type receptor 1 [Source:NCBI gene (formerly Entrezgene);Acc:102159820]
NOTCH3	notch receptor 3 [Source:NCBI gene (formerly Entrezgene);Acc:102158881]
AKAP6	A-kinase anchoring protein 6 [Source:VGNC Symbol;Acc:VGNC:85221]
OXTR	oxytocin receptor [Source:NCBI gene (formerly Entrezgene);Acc:397092]
-	prune homolog 2 with BCH domain [Source:NCBI gene (formerly Entrezgene);Acc:100153374]
DGKH	diacylglycerol kinase eta [Source:VGNC Symbol;Acc:VGNC:87273]
PCDH9	protocadherin 9 [Source:VGNC Symbol;Acc:VGNC:91217]
AR	androgen receptor [Source:NCBI gene (formerly Entrezgene);Acc:397582]
WWC1	WW and C2 domain containing 1 [Source:VGNC Symbol;Acc:VGNC:94986]
-	lysine acetyltransferase 6A [Source:VGNC Symbol;Acc:VGNC:95574]
ZNF652	zinc finger protein 652 [Source:VGNC Symbol;Acc:VGNC:99108]
CACNA1H	calcium voltage-gated channel subunit alpha1 H [Source:VGNC Symbol;Acc:VGNC:97041]
SYNPO2	synaptopodin 2 [Source:VGNC Symbol;Acc:VGNC:93672]
SFRP2	secreted frizzled related protein 2 [Source:VGNC Symbol;Acc:VGNC:92775]
MID1	midline 1 [Source:VGNC Symbol;Acc:VGNC:90213]
LOXL4	lysyl oxidase like 4 [Source:VGNC Symbol;Acc:VGNC:89783]
UBN2	ubinuclein 2 [Source:VGNC Symbol;Acc:VGNC:94664]
ADIPOQ	adiponectin, C1Q and collagen domain containing [Source:VGNC Symbol;Acc:VGNC:85140]
OLFML2B	olfactomedin like 2B [Source:VGNC Symbol;Acc:VGNC:91034]
STRA6	signaling receptor and transporter of retinol STRA6 [Source:VGNC Symbol;Acc:VGNC:93573]
RASSF4	Ras association domain family member 4 [Source:NCBI gene (formerly Entrezgene);Acc:100152580]
NOS1AP	chromosome 4 C1orf226 homolog [Source:NCBI gene (formerly Entrezgene);Acc:100620568]
SEL1L3	SEL1L family member 3 [Source:VGNC Symbol;Acc:VGNC:92688]
COL11A1	collagen type XI alpha 1 chain [Source:VGNC Symbol;Acc:VGNC:86862]

-	phospholipid-transporting ATPase IB [Source:NCBI gene (formerly Entrezgene);Acc:100625134]
NFATC1	nuclear factor of activated T cells 1 [Source:NCBI gene (formerly Entrezgene);Acc:397318]
OSR1	odd-skipped related transcription factor 1 [Source:VGNC Symbol;Acc:VGNC:91083]
TOX	thymocyte selection associated high mobility group box [Source:VGNC Symbol;Acc:VGNC:94322]
-	transmembrane protein 37 [Source:NCBI gene (formerly Entrezgene);Acc:100522166]
KAZN	kazrin, periplakin interacting protein [Source:NCBI gene (formerly Entrezgene);Acc:100520199]
THSD4	thrombospondin type 1 domain containing 4 [Source:VGNC Symbol;Acc:VGNC:93967]
PARM1	prostate androgen-regulated mucin-like protein 1 [Source:VGNC Symbol;Acc:VGNC:91182]
DES	desmin [Source:VGNC Symbol;Acc:VGNC:96159]
SDK1	sidekick cell adhesion molecule 1 [Source:VGNC Symbol;Acc:VGNC:92660]
-	-
-	gulonolactone (L-) oxidase [Source:NCBI gene (formerly Entrezgene);Acc:396759]
-	-
SATB1	SATB homeobox 1 [Source:VGNC Symbol;Acc:VGNC:92587]
-	DIX domain containing 1 [Source:VGNC Symbol;Acc:VGNC:99710]
NALCN	sodium leak channel, non-selective [Source:VGNC Symbol;Acc:VGNC:90572]
IGFBP5	insulin like growth factor binding protein 5 [Source:VGNC Symbol;Acc:VGNC:96368]
ABRAXAS1	abraxas 1, BRCA1 A complex subunit [Source:VGNC Symbol;Acc:VGNC:98910]
-	-
-	-
GDF6	growth differentiation factor 6 [Source:VGNC Symbol;Acc:VGNC:88402]
HSPB7	heat shock protein family B (small) member 7 [Source:NCBI gene (formerly Entrezgene);Acc:100627988]
-	steroid sulfatase [Source:NCBI gene (formerly Entrezgene);Acc:448816]
GLDN	gliomedin [Source:VGNC Symbol;Acc:VGNC:88479]
VIPR2	vasoactive intestinal peptide receptor 2 [Source:VGNC Symbol;Acc:VGNC:94828]
SEPTIN3	septin 3 [Source:VGNC Symbol;Acc:VGNC:92722]
-	-
-	-
5_8S_rRNA	5.8S ribosomal RNA [Source:RFAM;Acc:RF00002]
ssc-mir-421	ssc-mir-421 [Source:miRBase;Acc:MI0015925]
